# Initiation of migraine-related cortical spreading depolarization by hyperactivity of GABAergic neurons and Na_V_1.1 channels

**DOI:** 10.1172/JCI142203

**Published:** 2021-11-01

**Authors:** Oana Chever, Sarah Zerimech, Paolo Scalmani, Louisiane Lemaire, Lara Pizzamiglio, Alexandre Loucif, Marion Ayrault, Martin Krupa, Mathieu Desroches, Fabrice Duprat, Isabelle Léna, Sandrine Cestèle, Massimo Mantegazza

**Affiliations:** 1 Université Côte d’Azur and; 2 CNRS UMR7275, Institute of Molecular and Cellular Pharmacology (IPMC), Valbonne-Sophia Antipolis, France.; 3 Unità Operativa VII Clinical and Experimental Epileptology, Foundation IRCCS Neurological Institute Carlo Besta, Milan, Italy.; 4 Inria Sophia Antipolis Méditerranée, MathNeuro Project Team, Valbonne-Sophia Antipolis, France.; 5 Université Côte d’Azur, Laboratoire Jean-Alexandre Dieudonné, Nice, France.; 6 INSERM, Valbonne-Sophia Antipolis, France.

**Keywords:** Neuroscience, Genetic diseases, Neurological disorders, Sodium channels

## Abstract

Spreading depolarizations (SDs) are involved in migraine, epilepsy, stroke, traumatic brain injury, and subarachnoid hemorrhage. However, the cellular origin and specific differential mechanisms are not clear. Increased glutamatergic activity is thought to be the key factor for generating cortical spreading depression (CSD), a pathological mechanism of migraine. Here, we show that acute pharmacological activation of Na_V_1.1 (the main Na^+^ channel of interneurons) or optogenetic-induced hyperactivity of GABAergic interneurons is sufficient to ignite CSD in the neocortex by spiking-generated extracellular K^+^ build-up. Neither GABAergic nor glutamatergic synaptic transmission were required for CSD initiation. CSD was not generated in other brain areas, suggesting that this is a neocortex-specific mechanism of CSD initiation. Gain-of-function mutations of Na_V_1.1 (*SCN1A*) cause familial hemiplegic migraine type-3 (FHM3), a subtype of migraine with aura, of which CSD is the neurophysiological correlate. Our results provide the mechanism linking Na_V_1.1 gain of function to CSD generation in FHM3. Thus, we reveal the key role of hyperactivity of GABAergic interneurons in a mechanism of CSD initiation, which is relevant as a pathological mechanism of Na_v_1.1 FHM3 mutations, and possibly also for other types of migraine and diseases in which SDs are involved.

## Graphical abstract

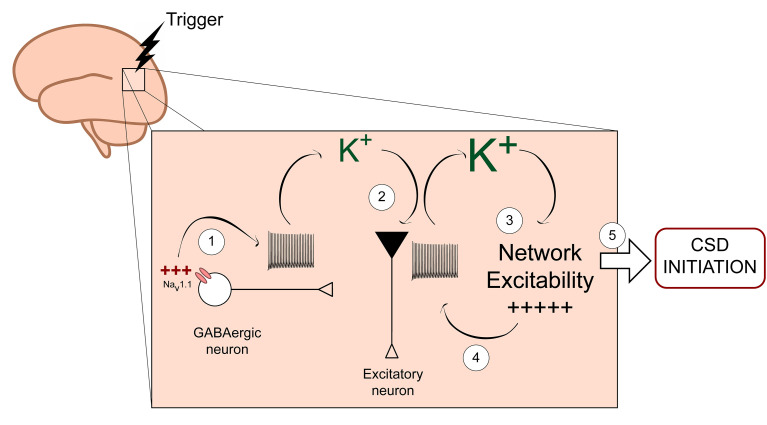

## Introduction

Spreading depolarizations (SDs) are waves of transient intense hyperexcitability of brain networks, which initiate focally and then slowly propagate, accompanied by modifications of ionic gradients and cell swelling (1, 2). SDs lead to long-lasting depolarization block of neuronal firing. SDs generated in normoxic conditions are implicated in migraine and epilepsy. SD-induced cortical spreading depression (CSD) of spontaneous activity causes migraine aura and could induce migraine headache by sensitization of meningeal nociceptors (2–5). SDs are observed after some types of epileptic seizures and have been implicated in sudden unexpected death in epilepsy (SUDEP) (6, 7). SDs generated in anoxic/hypoxic conditions are implicated in stroke, traumatic brain injury, and subarachnoid hemorrhage (1, 2, 8). SDs have been observed and extensively studied for decades, but specific pathological mechanisms that lead to their initiation and propagation are not clear, although it is hypothesized that glutamatergic activity plays a key role, in particular in CSD (1, 2).

A mendelian form of migraine with aura characterized by hemiparesis during the attacks, familial hemiplegic migraine (FHM) has become a model disease for more common forms and has allowed the identification of some molecular/cellular pathological mechanisms of human migraine and CSD (2, 3), which may be at least in part shared by SDs involved in other diseases. FHM type 1 (FHM1) is caused by gain-of-function mutations of the α1 subunit of the Ca_V_2.1 P/Q type Ca^2+^ channel (the *CACNA1A* gene; ref. 9), FHM type 2 (FHM2) is caused by loss-of-function mutations of the α2 subunit of the glial Na^+^/K^+^ pump (the *ATP1A2* gene; ref. 10). The experimental induction of CSD is facilitated in both FHM1 and FHM2 genetic mouse models (11, 12), primarily because of increased network excitability induced by excessive release or insufficient reuptake of glutamate (13–15), consistent with a similar overall mechanism affecting the glutamatergic system. These results have contributed to the current hypothesis that increased glutamatergic activity is the most important factor for triggering CSD.

FHM3 is caused by mutations of the Na_V_1.1 (*SCN1A*) Na^+^ channel (16), which is particularly important for GABAergic neuron excitability (17). Numerous epileptogenic Na_V_1.1 mutations have been identified, and studies performed both in vitro and in mouse models have shown that they cause loss of function of the channel, leading to decreased excitability of GABAergic neurons, reduced inhibition, and consequent hyperexcitability of neuronal networks and seizures (17–22). It has been proposed that Na_V_1.1 loss of function can facilitate brainstem SD induced by seizures, leading to SUDEP (7). We and others have provided evidence that, in contrast to epileptogenic mutations, FHM3 mutations cause gain of function of the channel, often increasing persistent current and inducing hyperexcitability of transfected GABAergic neurons in primary culture (21–30), which could be responsible for CSD initiation, as we have recently proposed in a computational model (31, 32). Interestingly, a recent work showed that knock-in mice carrying the FHM3 L263V Na_V_1.1 mutation experience spontaneous CSD events but not seizures, although detailed mechanisms of CSD generation have not been studied (33). Thus, these results point to different and counterintuitive mechanisms in CSD caused by Na_V_1.1 gain of function. However, there is not yet a causal link between Na_V_1.1 gain of function/GABAergic neuron hyperactivity and CSD, and it is not clear whether and how CSD (and possibly other SDs) could be generated by these dysfunctions.

Here, we addressed these issues inducing acute Na_V_1.1 gain of function that mimics the functional effect of FHM3 mutations and performing selective optogenetic stimulation of GABAergic neurons, carrying out experiments in vivo, in brain slices and in cell lines.

## Results

### Acute gain of function of NaV1.1 channels induces CSD selectively in the neocortex.

To disclose the role of the Na_V_1.1 channels’ gain of function in the generation of CSD, we used the spider toxin Hm1a that has been reported as a specific Na_V_1.1 enhancer (34). We confirmed, by performing whole-cell patch-clamp recordings of Na^+^ currents in cell lines, that in our conditions Hm1a selectively targets Na_V_1.1 over the 2 other Na_V_ isoforms expressed in the adult cortex, Na_V_1.2 and Na_V_1.6, although only at low concentration (10 nM; Supplemental Figure 1; supplemental material available online with this article; https://doi.org/10.1172/JCI142203DS1). Importantly, Hm1a induced a 12-fold increase of persistent current, an effect that is comparable to that previously observed with FHM3 mutations (24, 25, 28–30), making it a good pharmacological tool for modeling the effect of these mutations.

**Figure 1:**
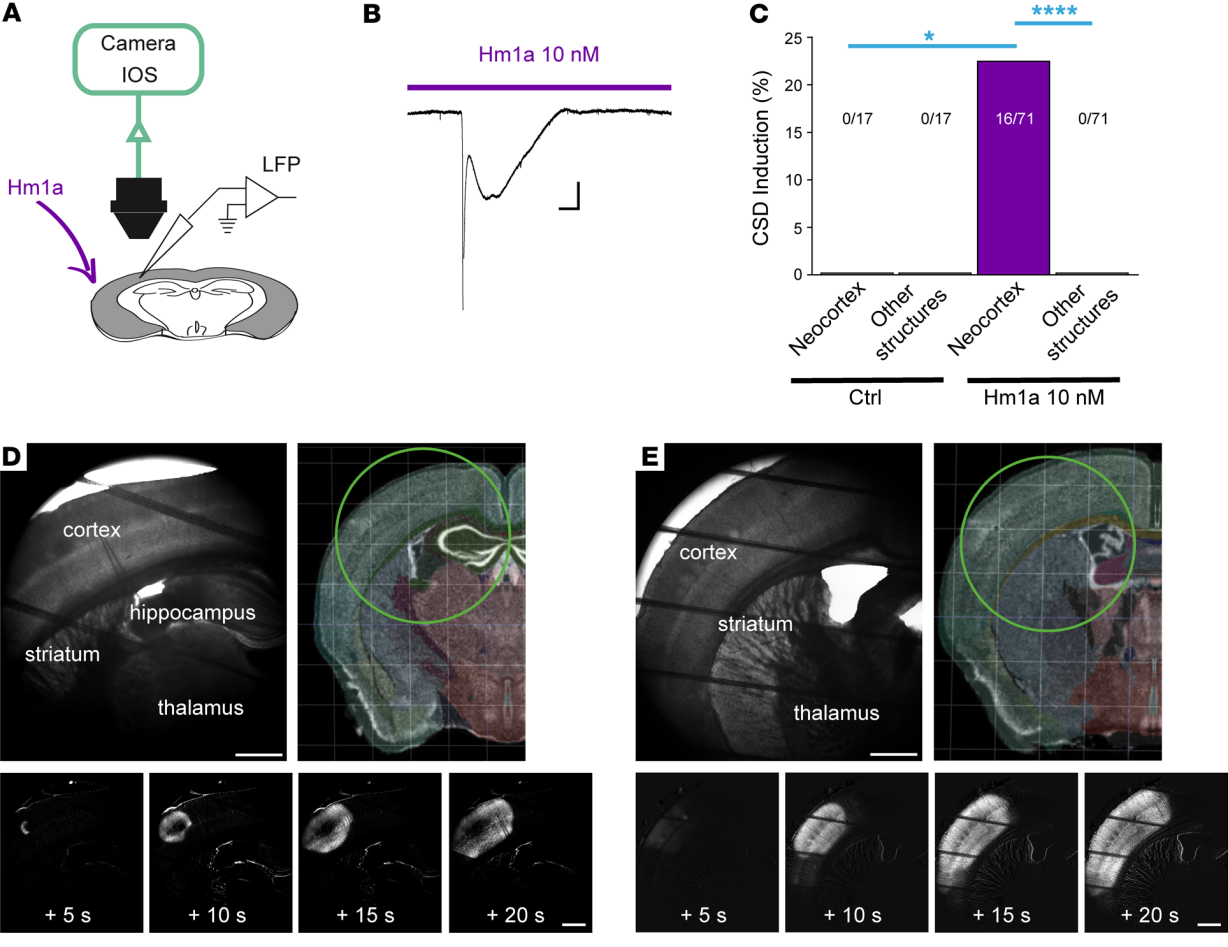
The selective Na_V_1.1 enhancer Hm1a specifically triggers CSD in the neocortex. (**A**) Experimental setting: brain slices were perfused with 10 nM Hm1a, a concentration at which Hm1a is selective for Na_V_1.1 (Supplemental Figure 1), and CSD induction was monitored with extracellular LFP recordings and IOS imaging obtained in extended brain regions (4× objective, 0.35× camera adapter). (**B**) Representative LFP recording of a CSD observed in the neocortex during the perfusion with Hm1a. Scale bars: 1 mV, 20 seconds. (**C**) Overall results showing the lack of spontaneous CSD in control (0/17 slices), and success rate for neocortical induction with bath application of Hm1a (16/71), which never triggered CSD in other structures (Fisher’s exact test, Bonferroni correction, neocortex control versus neocortex Hm1a, *P = 0.04; neocortex Hm1a versus other structures’ Hm1a, ****P = 10^–4^). (**D**) Upper left panel, raw transmitted light image of a representative coronal slice including the neocortex, the hippocampus, the dorsal striatum, the globus pallidus, and the thalamus; upper right panel, illustration of a whole hemisphere (Brain Explorer, Allen Institute) in which the imaged area is indicated by the circle (see Supplemental Figure 2 for additional details). The 4 bottom panels correspond to time series of image processing of IOS acquisitions (one image every 5 seconds, the first one 5 seconds after CSD initiation; see Methods), which show that CSD was triggered only in the neocortex. Scale bar: 500 μm. (**E**) Raw transmitted light image of another representative coronal slice including the neocortex, the dorsal striatum, the globus pallidus, and the thalamus (upper left); illustration of a whole hemisphere (Brain Explorer, Allen Institute) in which the imaged area is indicated by the circle (see Supplemental Figure 2 for additional details). The 4 bottom panels are a time series of processed IOS images, which show that CSD was triggered only in the neocortex. Scale bar: 500 μm (see Supplemental Video 1).

We tested the effect of the toxin in whole-brain slices that included different structures, performing extracellular local field potential (LFP) recordings and intrinsic optical signal (IOS) imaging in an extended area (Figure 1 and Supplemental Figure 2). Bath application of 10 nM Hm1a lead to spontaneous CSD ignition, which was observed both as DC shift in LFP recordings (Figure 1B) and as propagating wave in IOS images (Figure 1, D and E and Supplemental Video 1). CSD was elicited in 22.5% of the slices within 10 minutes of Hm1a application (our time limit for determining successful induction), whereas we have never observed CSD in control conditions (Figure 1C). Interestingly, CSD was elicited only in the neocortex and never in the other structures monitored (hippocampus, dorsal striatum, globus pallidus, and thalamus; Figure 1, D and E and Supplemental Video 1). To confirm that this was a neocortex-specific effect of Hm1a, we evaluated whether in our conditions other brain areas were able to generate SDs by applying short puffs of 130 mM KCl, a classical method of CSD induction (2, 35). The success rate for CSD induction was 100% in all the structures tested: the neocortex (Supplemental Figure 3, A–C and Supplemental Video 2), the striatum (Supplemental Figure 3D_1_ and Supplemental Video 3), and the hippocampus (Supplemental Figure 3D_2_ and Supplemental Video 4). Neither Hm1a-induced nor KCl-induced CSD propagated outside the structures in which they were induced.

**Figure 2:**
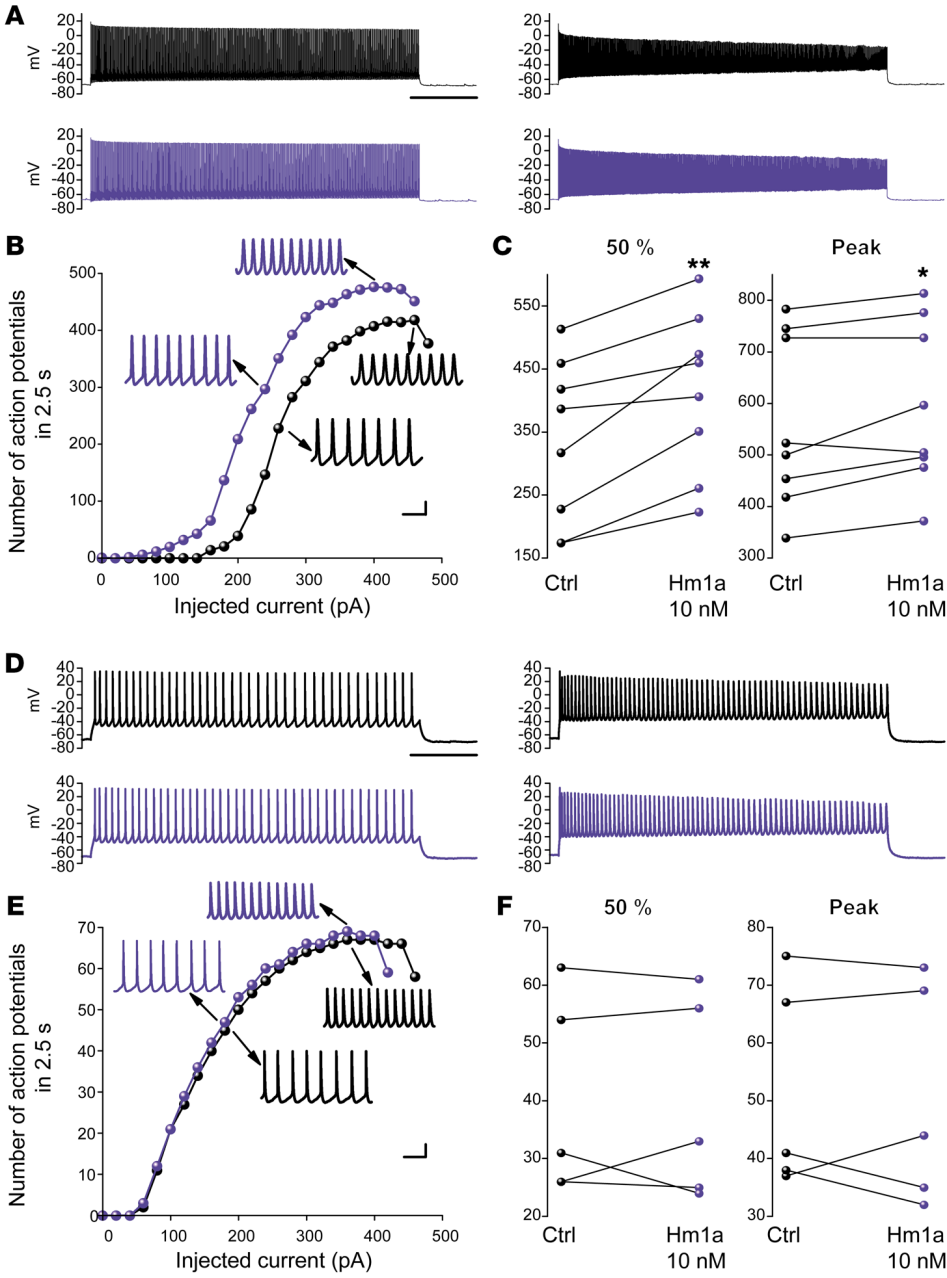
Hm1a increases the excitability of fast-spiking GABAergic neurons but not of pyramidal neurons. Recordings in neocortical layer II–III of GAD67-GFP knock-in mice (which label GABAergic neurons). (**A**) Left, representative traces at 50% of the input-output relationship, recorded from a fast-spiking neuron before (black) and during (violet) perfusion with 10 nM Hm1a. Scale bar: 500 ms. Right, representative traces recorded from the same interneuron at the peak of the input-output relationship. (**B**) Representative plot of the effect of Hm1a on the input-output relationship recorded from the same fast-spiking interneuron, and magnified traces taken from a 50 ms time window at the middle of the traces displayed in **A**. Scale bars: 20 mV, 10 ms. (**C**) Firing frequency for each recorded neuron before and during Hm1a perfusion at 50% (mean increase Δ = 28.4% ± 7.0%, ** *P =* 0.008; median = 352AP/2.5s, mean ± SEM = 334 ± 46 in control; 433, 412 ± 45 with Hm1a) and at the peak of the input-output relationship (mean increase Δ = 7.1% ± 2.6%; *P = 0.03; 511 AP/2.5s, 561 ± 59 in control; 551, 595 ± 57 with Hm1a) (*n =* 8). (**D**) Left, representative traces at 50% of the input-output relationship, recorded from a pyramidal neuron before (black) and during (violet) perfusion with Hm1a. Scale bar: 500 ms. Right, representative traces recorded from the same pyramidal neuron at the peak of the input-output relationship, before (black) and during (violet) perfusion with Hm1a. (**E**) Representative input-output relationship obtained from the same pyramidal neuron and magnified traces taken from a 50 ms time window in the middle of the traces displayed in **D**. Scale bars: 20mV, 100ms. (**F**) Firing frequency for each recorded neuron before and during Hm1a perfusion at 50% (Δ = –2.2% ± 6.4%, *P =* 1; median = 31AP/2.5s, mean ± SEM = 40.0 ± 7.7 in control; 33, 39.8 ± 7.8 with Hm1a) and at the peak of the input-output relationship (Δ = 0.2% ± 7.9%, *P =* 0.9; 41AP/2.5s, 51.6 ± 8.0 in control; 44, 50.6 ± 8.6 with Hm1a) (*n =* 5). Wilcoxon’s signed ranks test for all the comparisons.

We performed current-clamp patch-clamp recordings from both GABAergic and pyramidal neurons in neocortical Layer II-III of brain slices from GAD67-GFP knock-in mice (which selectively label GABAergic neurons), to confirm that the Hm1a-induced gain of function of Na_V_1.1 can increase excitability preponderantly of GABAergic neurons in the neocortex. We maintained the resting membrane potential at around –70mV and elicited action potential discharges with injections of 2.5-seconds-long depolarizing current steps of increasing amplitude, comparing the properties of input-output relationships before and after 10 minute perfusion with 10 nM Hm1a. In order to minimize the variability of firing patterns of GABAergic neurons, we selected fast-spiking nonadapting neurons for the analysis (Figure 2, A–C), which have firing features typical of parvalbumin positive (PV+) neurons. All the recorded pyramidal neurons showed regular spiking discharges (Figure 2, D–F). The application of Hm1a induced a leftward shift of the input-output relationship (28% mean increase of firing frequency at 50% of the input-output) and an increase of the maximal firing frequency (7% mean increase) in GABAergic neurons, whereas the firing properties of pyramidal glutamatergic neurons were not modified.

Thus, Hm1a induces in the neocortex hyperexcitability of GABAergic neurons but not of pyramidal neurons, and specifically triggers CSD in the neocortex, although in our experimental conditions SDs can be induced by puffs of KCl in all the structures tested (neocortex, hippocampus, and striatum).

### Optogenetic hyperactivation of GABAergic neurons can initiate CSD selectively in the neocortex.

**Figure 3:**
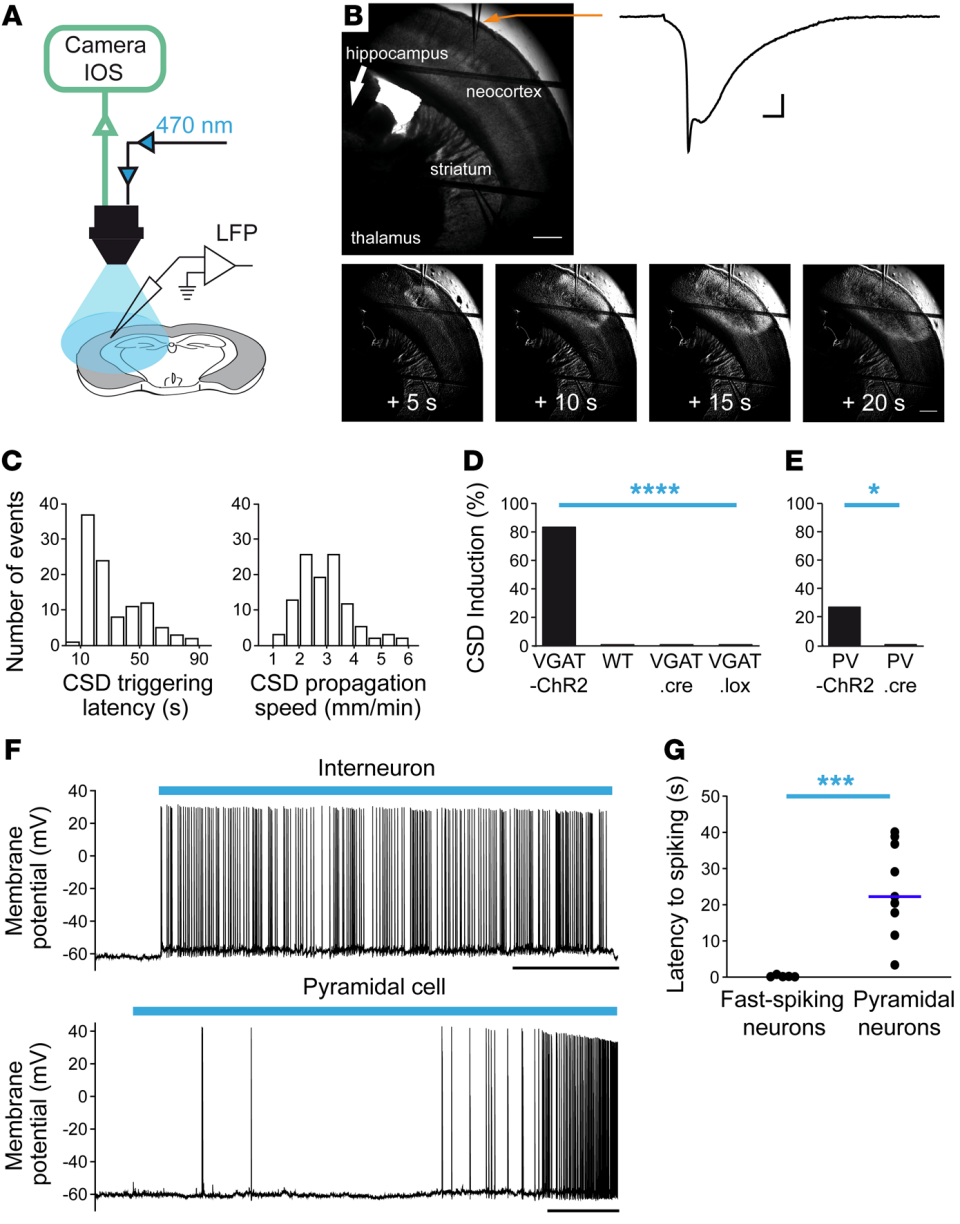
CSD is triggered specifically in the neocortex by optogenetic-induced hyperactivity of GABAergic neurons. (**A**) Experimental setting for optogenetic stimulations of a complete hemisphere in coronal slices (the area of illumination was larger than the area of image acquisition). (**B**) Representative CSD that was induced only in the neocortex in slices from VGAT-ChR2 mice containing neocortex, hippocampus, dorsal striatum, and thalamus, revealed by both the negative DC shift in the LFP and the IOS propagating wave. The 4 bottom panels are a time series corresponding to image-processed IOS acquisitions (one image every 5 seconds, the first one 5 seconds after CSD initiation; see Methods). Scale bars: 500 μm. (**C**) Distribution of latencies of CSD initiation upon 470 nm illumination in VGAT-ChR2 slices (median *=* 19 seconds, *n =* 103 slices) and distribution of propagation speed of optogenetic-induced CSD in VGAT-ChR2 slices (median *=* 3.18 mm/min, *n =* 103 slices). (**D**) Success rate of optogenetic CSD obtained in a different series of experiments comparing slices from VGAT-ChR2 (11/13 slices), WT (0/14), VGAT.Cre (0/10), and ChR2.lox (0/10) mice (Fisher’s exact test, ****P = 7 10^–5^). (**E**) Success rate of optogenetic CSD obtained in slices from PV-ChR2 mice (4/14 slices) or control PV-cre littermates (0/15 slices) (Fisher’s exact test, *P = 0.04). (**F**) Representative whole-cell patch-clamp recordings of GABAergic and pyramidal neurons in layer II–III upon optogenetic illumination: a fast-spiking GABAergic neuron responded to the 470 nm illumination (blue bar) with short latency, whereas a pyramidal neuron responded with longer latency. Scale bar: 20 seconds. (**G**) Overall latencies to spiking during 470 nm illumination for fast-spiking neurons (median *=* 0.30 seconds; *n =* 5) and pyramidal neurons (median *=* 22.26 seconds; *n =* 12) (Mann-Whitney test, ***P = 0.001). These recordings were not performed at the site of initiation, which, for this experimental setting, was variable within the neocortex and not identifiable a priori.

To directly investigate the role of GABAergic neurons, we used hemizygous VGAT.cre-ChR2(H134R)tdTomato.lox (VGAT-ChR2) mice, in which channelrhodopsin (ChR2)–H134R is selectively expressed in these neurons in different brain regions (Supplemental Figure 4 and ref. 36). We activated GABAergic neurons in coronal brain slices, illuminating an entire cerebral hemisphere with blue light, and we monitored CSD generation by both LFP recordings and IOS imaging of several brain structures (Figure 3, A and B and Supplemental Video 5). Notably, similar to the experiments performed with Hm1a, the optogenetic activation of GABAergic interneurons induced CSD only in the neocortex. We never observed it in the hippocampus, striatum, or thalamus. Mean latency to induction was 26.2 ± 1.8 seconds (*n =* 104) and propagation speed was 3.2 ± 0.1 mm/min (*n =* 104; Figure 3C). Macroscopic features of CSD induced by optogenetic stimulation were similar to those of CSD triggered by a focal puff of 130 mM KCl in the neocortex (Supplemental Figure 3, A–C and Supplemental Video 2) or by perfusion with Hm1a (Figure 1 and Supplemental Video 1). We performed a subset of experiments for determining the rate of success of optogenetic CSD induction (Figure 3D; see Methods for detailed procedure), observing that it was induced in 85% of VGAT-ChR2 slices (always only in the neocortex) and never with control littermates (i.e., slices from VGAT-Cre, ChR2.lox, or WT mice). Notably, CSD was readily induced by focal puff of 130 mM KCl in all the slices, both from VGAT-ChR2 mice and control littermates (Supplemental Figure 3C), confirming that all the slices could generate CSD (not only those from VGAT-ChR2 mice). Moreover, as already highlighted before, CSD was readily induced by focal puff of 130 mM KCl also in the hippocampus and the striatum (Supplemental Figure 3D). Several studies in different models have shown that functions of PV+ GABAergic neurons are significantly modified by *SCN1A* mutations (21, 22), FHM3 mutations can induce hyperexcitability of these neurons, as observed in the accompanying paper by Auffenberg et al. (37), and we have quantified the effect of Hm1a on these neurons (Figure 2). Notably, we were able to induce optogenetically neocortical CSD also in slices from hemizygous PV.cre-ChR2(H134R)tdTomato.lox (PV-ChR2) mice (Figure 3E), in which ChR2-H134R is selectively expressed in PV+ neurons, although with a lower success rate (28%) than in VGAT-ChR2 slices. Thus, CSD initiation by overactivation of GABAergic neurons is a neocortex-specific mechanism, similarly to CSD initiation by Hm1a, and overactivation of a GABAergic subpopulation (PV+ neurons) is sufficient for its induction.

We then evaluated the response of pyramidal and GABAergic neurons in layer II–III of the neocortex of VGAT-ChR2 mice to the optogenetic stimulation before the induction of CSD, to validate the specific response of GABAergic neurons to the illumination, performing whole-cell patch-clamp recordings (Figure 3F). We observed that GABAergic neurons (which we identified as nonpyramidal fast-spiking nonadapting neurons) directly responded with short latency to the illumination, which triggered long-lasting firing, whereas pyramidal neurons did not respond directly to the illumination and begun to spike after few tens of seconds of illumination (Figure 3G), consistent with a specific rapid and long-lasting activation of GABAergic neurons.

**Figure 4:**
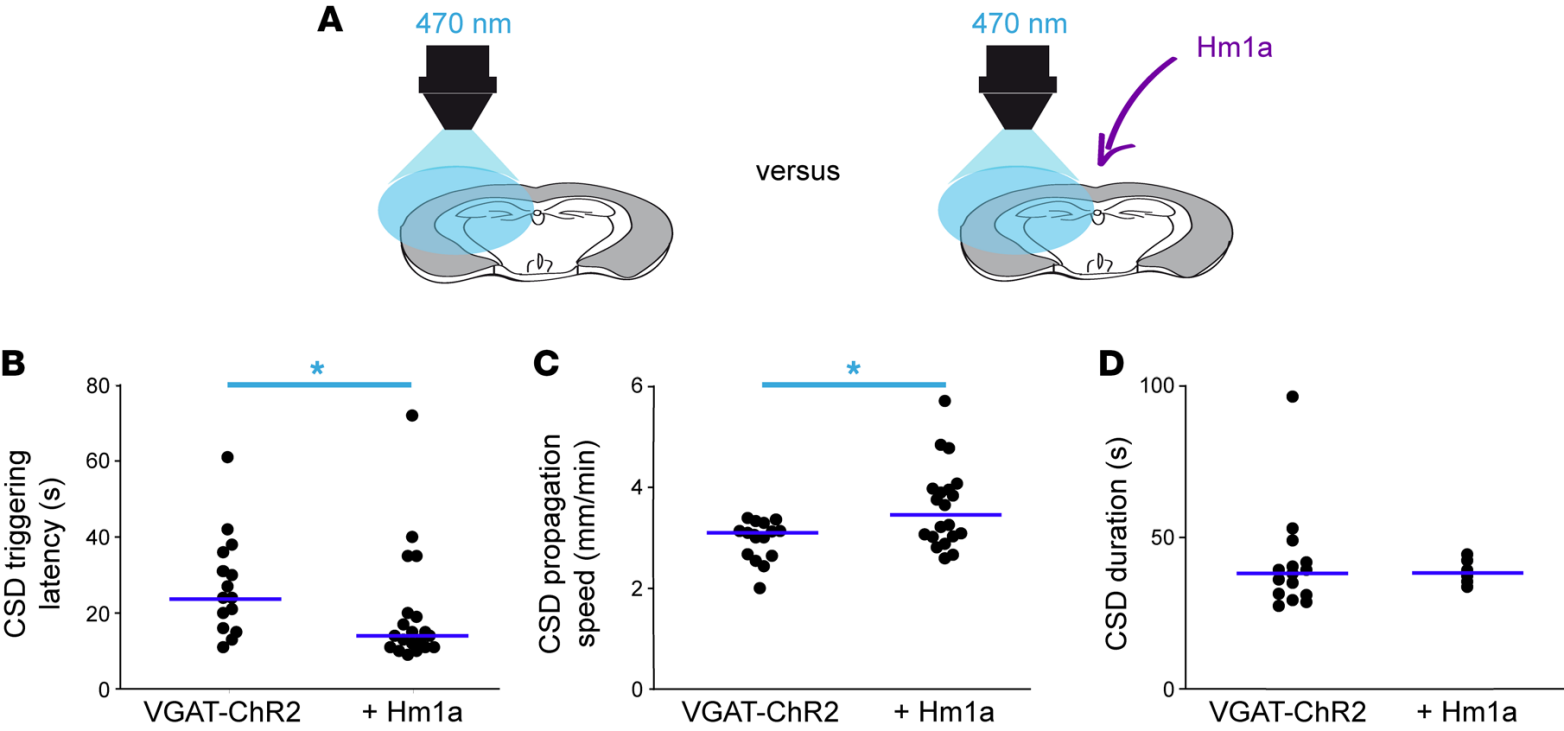
Effect of Hm1a on optogenetic CSD induction. (**A**) Further series of experiments in which features of optogenetic CSD induction were compared in VGAT-ChR2 slices perfused with Hm1a (but in which the toxin did not induce CSD within 10 minutes) and control VGAT-ChR2 slices, waiting 10 minutes to illuminate (these experiments are included in Figure 1D). (**B**) Latencies of optogenetic CSD measured in control VGAT-ChR2 slices (median *=* 24 seconds, mean ± SEM = 27.3 ± 3.4 seconds; *n =* 15 slices) and VGAT-ChR2 slices perfused with Hm1a (median *=* 14 seconds, mean ± SEM = 19.8 ± 3.4 seconds; *n =* 20 slices) (Mann-Whitney test, *P = 0.014). (**C**) Propagation speed of optogenetic CSD in the same slices (control VGAT-ChR2, median *=* 3.14 mm/min, mean ± SEM = 3.0 ± 0.1 mm/min, *n =* 15 slices; VGAT-ChR2 slices perfused with Hm1a without CSD, median *=* 3.50 mm/min, mean ± SEM = 3.6 ± 0.2 mm/min, *n =* 20 slices) (Mann-Whitney test, *P = 0.025). (**D**) Duration of optogenetic-induced CSD measured at half width of the LFP DC shift (control VGAT-ChR2 slices, median *=* 38.1 seconds, mean ± SEM = 41.1 ± 4.4 seconds, *n =* 15 slices, VGAT-ChR2 slices perfused with Hm1a but without CSD, median *=* 38.3 seconds, mean ± SEM = 38.7 ± 1.7 seconds, *n =* 6 slices) (Mann-Whitney test, *P =* 0.7).

In a further series of experiments, we evaluated the effect of Hm1a on optogenetic-induced CSD, hypothesizing a synergic role (Figure 4). In fact, in brain slices in which Hm1a application did not induce CSD, subsequent optogenetic stimulation induced CSD with a 28% reduction of triggering latency and a 20% increase of propagation speed, control slices without Hm1a. CSD was triggered only in the neocortex in this series of experiments and its duration, evaluated measuring the LFP half-width, was not modified by Hm1a. Therefore, optogenetic CSD was facilitated by Hm1a, confirming the key role of the Na_V_1.1 channels’ gain of function and overactivation of GABAergic neurons in the mechanism of CSD initiation that we have disclosed.

### Computational model of CSD initiation by overactivation of Na_V_1.1 channels and GABAergic neurons.

We have recently shown that, in simulations obtained with a conductance-based model of a GABAergic neuron connected to a pyramidal neuron, an overactivation of the GABAergic neuron can lead to depolarizing block of the pyramidal neuron, which we considered the initiation of CSD (31). In that model, the overactivation of the GABAergic neuron was obtained, increasing its external depolarizing input (modeling a pathological state, as well as a condition similar to our optogenetic stimulation). Several putative GABAergic activation-related mechanisms were tested, and we identified the frequency of interneuron firing and the related increase of [K^+^]_out_ as the key element for inducing depolarizing block of the pyramidal neuron (31).

**Figure 5:**
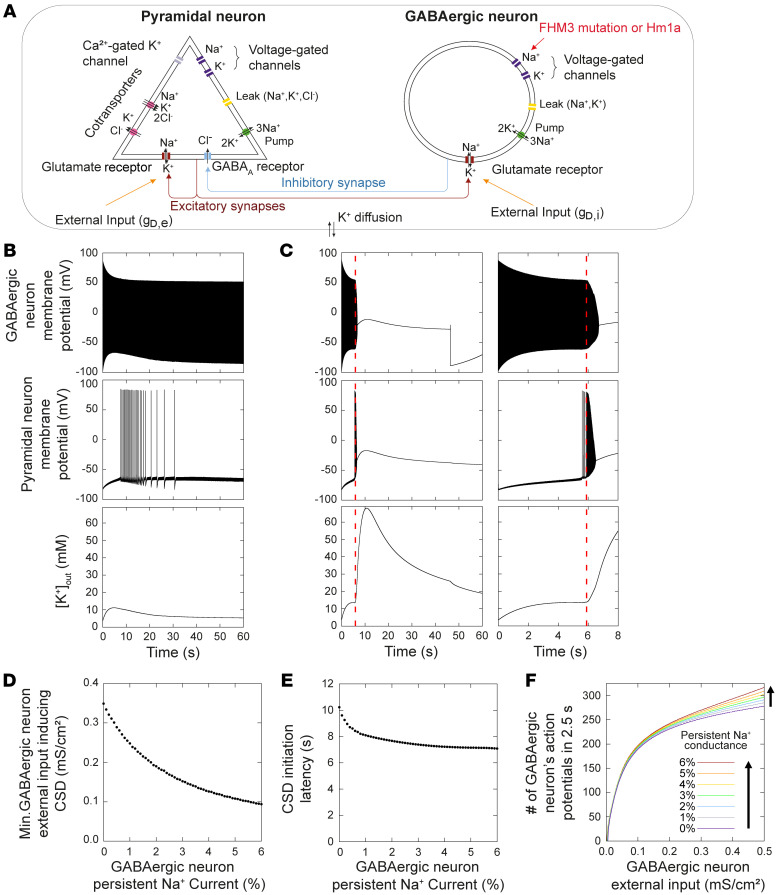
The increase of GABAergic neuron’s persistent current facilitates the initiation of CSD in a computational model. (**A**) Diagram illustrating the conductance-based computational model that we used to model the effect of migraine mutations and of Hm1a by increasing the persistent Na^+^ current of the GABAergic neuron. *gD,e* and *gD,i* are glutamatergic conductances that model the baseline excitatory inputs (excitatory drive) of the pyramidal and of the GABAergic neuron, respectively. (**B**) Simulation, with GABAergic neuron’s physiologic persistent Na^+^ current (1%), of the effect of a constant external depolarizing input to the GABAergic neuron (*gD,i* = 0.1 mS/cm^2^) without input to the pyramidal neuron (*gD,e* = 0 mS/cm^2^): membrane potential of the GABAergic neurons (upper), membrane potential of the pyramidal neuron (middle), and extracellular K^+^ concentration (lower). (**C**) Same simulation with increased GABAergic neuron’s persistent Na^+^ current (6%, mimicking the effect of FHM3 mutations and of Hm1a); the right panels display with an enlarged time scale the first phase of the simulation shown in the panels on the left. The vertical dotted line indicates the beginning of the large [K^+^]_out_ increase that leads to depolarizing block. (**D**) Effect of an increase of the GABAergic neuron’s persistent Na^+^ current on the lowest external input to the GABAergic neuron (*gD,i*) sufficient to induce depolarizing block. (**E**) Effect of an increase of the GABAergic neuron’s persistent Na^+^ current on the latency of depolarizing block with *gD,i* = 0.349 mS/cm^2^ (the lowest input to the GABAergic neuron able to generate CSD with 0% persistent Na^+^ conductance; see panel **D**). (**F**) Effect of the amount of persistent current on the firing frequency of the GABAergic neuron, in a simulation in which the pyramidal neuron was removed, reflecting the direct effect of the persistent current on the firing properties of the GABAergic neuron.

Here, we have refined the model, in particular improving the features of the GABAergic neuron, which now better models a fast-spiking cortical interneuron (38), and including complete dynamics of ion concentrations for both neurons (Figure 5A; see Methods), so that the modifications of ion concentrations can modulate the activity of both neurons. The direct overactivation of the GABAergic neuron generated simulations (not shown) that were similar to those obtained in our previous study, under similar conditions (31). We tested the effect of an increase of the persistent Na^+^ current of the GABAergic neuron, which mimics the common effect of most FHM3 mutations (21, 23–30; see also the accompanying paper by Auffenberg et al. [ref. 37]), as well as that of Hm1a. We modeled the control physiological condition implementing a persistent conductance equal to 1% of the maximal Na^+^ conductance, and we increased it up to 6% to simulate a pathological condition or the presence of Hm1a (which is conservative in comparison with the quantitative effect of FHM3 mutations or Hm1a). In the physiological condition (Figure 5B), an external depolarizing input to the GABAergic neuron (*gD,i*) of 0.1 mS/cm^2^, without application of external input to the pyramidal neuron (*gD,e*), was able to induce high frequency firing of the GABAergic neuron. As previously shown, long-lasting, high-frequency firing of the GABAergic neuron overcame its inhibitory effect because it increased [K^+^]_out_ (31). In the simulation of Figure 5B, [K^+^]_out_ reached a maximum of 11.2 mM, depolarizing the pyramidal neuron and triggering its spiking, which was transient and ended after few seconds, when [K^+^]_out_ relaxed to lower levels. In this condition, there was no depolarizing block (CSD initiation). When the same simulation was run with persistent Na^+^ conductance of the GABAergic neuron increased to 6% (Figure 5C), in the initial phase the GABAergic neuron discharged at higher frequency leading to a larger increase of [K^+^]_out_ (up to 13.6 mM). Importantly when the pyramidal neuron was engaged in firing, both neurons underwent depolarizing block, with [K^+^]_out_ rising to greater than 60 mM, indicating CSD initiation. In fact, the increase of persistent Na^+^ current of the GABAergic neuron facilitated CSD, leading to a decrease of the minimal *gD,i* that can induce CSD initiation (Figure 5D). Moreover, similar to the experimental data of Figure 4B, the increase of persistent current lead to a reduction of the CSD initiation latency (Figure 5E); CSD was induced in this simulation with *gD,i* equal to 0.349 mS/cm^2^, which was the minimal input able to induce CSD with 0% persistent current (Figure 5D).

Finally, in simulations in which the pyramidal neuron was removed from the model, we evaluated the effect of an increase of persistent current on the “intrinsic” firing frequency of the GABAergic neuron. We observed that it was increased at all the levels of external input tested (Figure 5F). This shows that the persistent current can increase the firing frequency of the GABAergic neuron independently from the interactions with the pyramidal neuron (e.g., synaptic input and modifications of ion concentrations).

Overall, the model shows that an increase of persistent Na^+^ current, mimicking FHM3 mutations or Hm1a, induces hyperexcitability of the GABAergic neuron, leading to a facilitation of CSD, which is ignited with lower values of external input and shorter latency and is consistent with the experimental data. Interestingly, a prediction of the model obtained in the simulations presented here and elsewhere (31), is that the key factor for CSD initiation induced by overactivation of GABAergic neurons is an increase of [K^+^]_out_, initially directly generated by the spiking of the GABAergic neuron. Thus, our experimental system investigated the detailed mechanisms of CSD initiation.

### Detailed mechanisms of CSD initiation.

**Figure 6:**
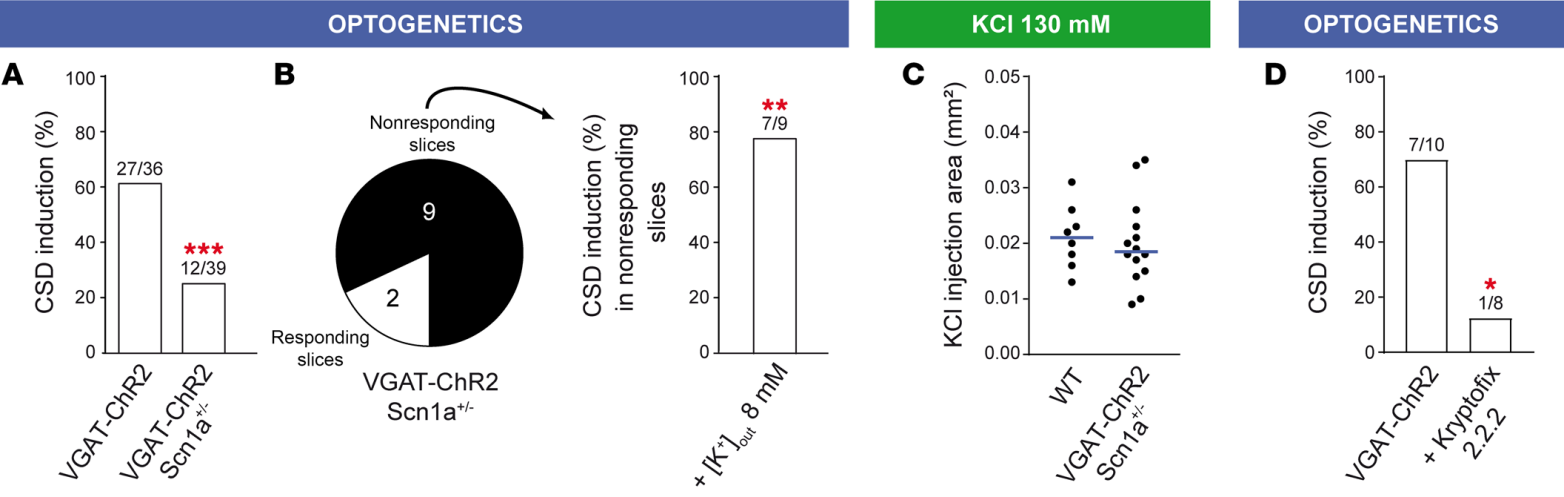
Effect of the reduction of GABAergic neuron excitability and extracellular K^+^ chelation on optogenetic CSD induction. (**A**) Reduction of optogenetic CSD induction success rate in slices from VGAT-ChR2-*Scn1a*^+/–^ mice compared with slices of VGAT-ChR2 littermates (Fisher’s exact test, ***P = 0.0002). (**B**) A different series of experiments in which optogenetic CSD was induced after the increase of [K^+^]_out_ to 8 mM in VGAT-ChR2-*Scn1a*^+/–^ slices, and in which optogenetic CSD was not previously induced with standard [K^+^]_out_ (Fisher’s exact test, **P = 0.002). (**C**) Threshold of CSD induction quantified by the area of puff applications of 130 mM KCl in slices from VGAT-ChR2-*Scn1a*^+/–^ (median = 0.021, mean ± SEM = 0.021 ± 0.002 mm^2^, *n =* 14) compared with VGAT-ChR2 littermates (0.019; 0.020 ± 0.002 mm^2^, *n =* 8; Mann-Whitney test, *P =* 0.58). (**D**) Reduction of optogenetic CSD induction rate with extracellular K^+^ chelation by application of 2.2.2-cryptand (Kryptofix2.2.2): success rate 70% (*n =* 10) in control VGAT-ChR2 slices, 12.5% (*n =* 8) with 2 mM Kryptofix2.2.2 (Fisher’s exact test, *P = 0.025).

We initially evaluated the effect of Na_V_1.1 loss of function on CSD initiation, to compare it with the gain of function tested above. We crossed VGAT-ChR2 mice with knock-out *Scn1a*^+/–^ mice, which model the epileptic encephalopathy Dravet syndrome and in which one allele of the *Scn1a* gene is not functional, causing Na_V_1.1 haploinsufficiency and hypoexcitability of GABAergic neurons (17, 21). This selective effect on GABAergic neurons has been observed in numerous studies, also at the age that we have used for our investigation, although there could be remodeling at later developmental stages (39). In brain slices from VGAT-ChR2/*Scn1a*^+/–^ mice, the success rate of optogenetic CSD induction was reduced 2.4-fold (Figure 6A), showing that Na_V_1.1 loss of function and reduced excitability of GABAergic neurons can inhibit CSD initiation by optogenetic activation of GABAergic neurons. Then, we tested the importance of extracellular K^+^ accumulation as a key parameter for CSD initiation. We initially used VGAT-ChR2/*Scn1a*^+/–^ slices in which the optogenetic stimulation did not trigger CSD, perfusing them with rACSF in which [K^+^] was moderately increased (from 3.5 to 8 mM), and applying a second optogenetic stimulation. Notably, we found that the reduced success rate of CSD induction in VGAT-ChR2/*Scn1a*^+/–^ slices was rescued with 8 mM [K^+^]_out_ (Figure 6B). The latency of optogenetic CSD induction was not different in the 3 conditions (median 39 seconds, mean ± SEM 39 ± 4 seconds for control VGAT-ChR2, *n =* 23, 4 slices not included because initiation was outside the imaged area; 38 seconds, 45 ± 5 seconds for VGAT-ChR2/*Scn1a*^+/–^, *n =* 11, 1 slice not included; 47 seconds, 51 ± 14 seconds for VGAT-ChR2/*Scn1a*^+/–^ with 8 mM KCl, *n =* 6, 1 slice not included; *P =* 0.70 Kruskal-Wallis test), and there was a trend toward an increase of the propagation speed in VGAT-ChR2/*Scn1a*^+/–^ slices (median 3.07 mm/min, mean ± SEM 2.41 ± 0.15 mm/min for control VGAT-ChR2, *n =* 27; 3.12 mm/min, 3.2 ± 0.2 mm/min for VGAT-ChR2/*Scn1a*^+/–^, *n =* 12; 3.95 mm/min, 3.97 ± 0.28 mm/min for VGAT-ChR2/*Scn1a*^+/–^ with 8 mM KCl, *n =* 7; *P =* 0.07 Kruskal-Wallis test). Importantly, the threshold of CSD induced applying puffs of 130 mM KCl (obtained by quantifying the area of KCl application as in ref. 35) was not different in slices from VGAT-ChR2/*Scn1a*^+/–^ and VGAT-ChR2 mice (Figure 6C), confirming that the inhibition of optogenetic CSD in VGAT-ChR2/*Scn1a*^+/–^ slices is not caused by a generic reduced propensity to CSD generation caused by modifications induced by the epileptic condition. Notably, CSD propagation speed was increased (median 4.32 mm/min, mean ± SEM 4.39 ± 0.29 mm/min, *n =* 14) compared with VGAT-ChR2 littermates (3.02 mm/min, 3.27 ± 0.35mm/min, *n =* 8; *P =* 0.047 Mann-Whitney test), consistent with the trend observed in optogenetic experiments and with different mechanisms of initiation and propagation.

**Figure 7:**
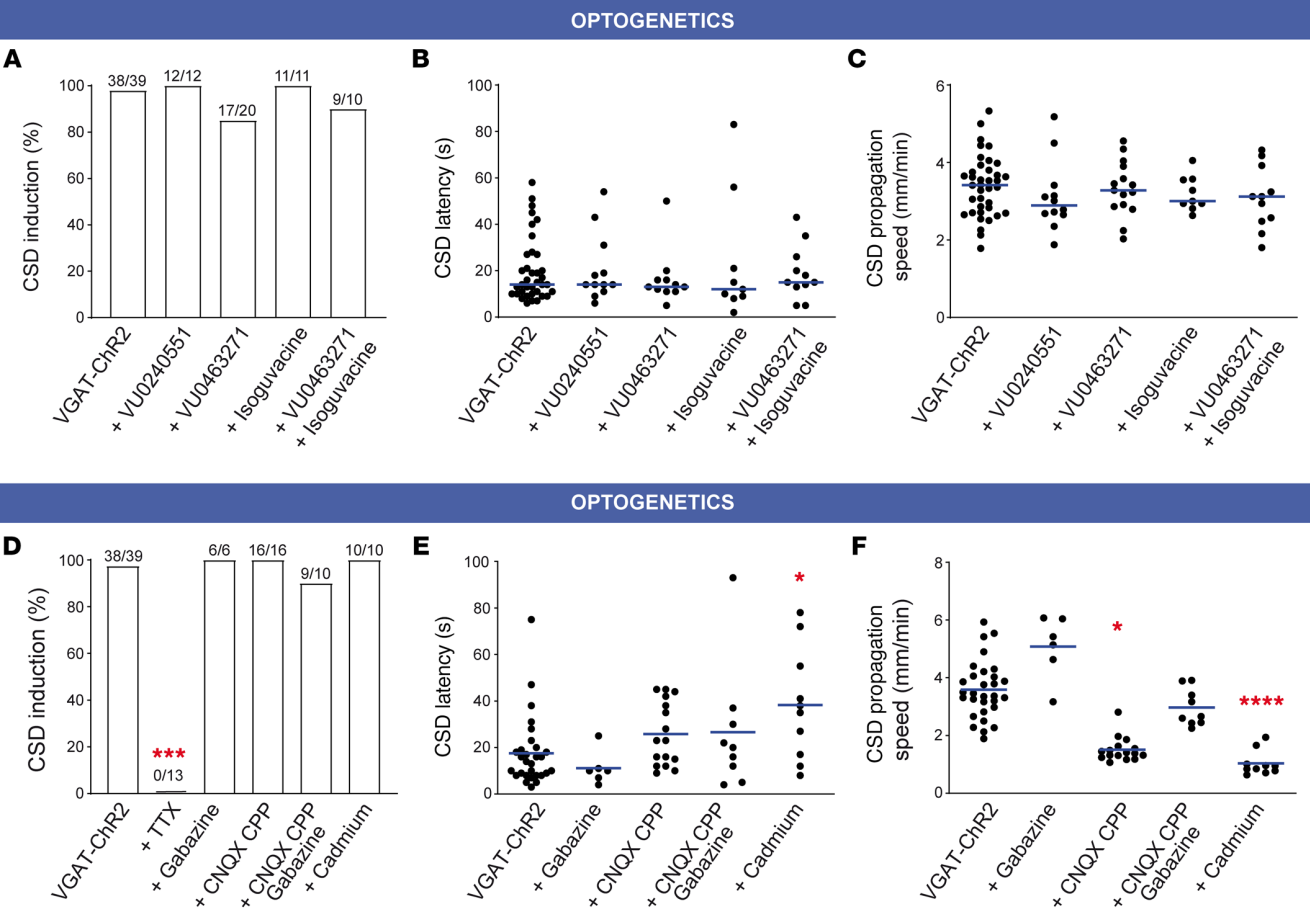
Effect of block of KCC2, neuronal excitability, or synaptic transmission on optogenetic CSD induction. (**A**) Success rate of optogenetic CSD in VGAT-ChR2 slices in control (100%, *n =* 39) with the KCC2 blocker VU0240551 10 μM (100%, *n =* 12), the KCC2 blocker VU04663271 10 μM (85%, *n =* 20), the GABA-A agonist Isoguvacine 10 μM (100%, *n =* 11), or VU0463271 10 μM + Isoguvacine 10 μM (90%, *n =* 10); *P =* 0.0493 overall Fisher’s exact test, not significant with Bonferroni-corrected pairwise test. (**B**) Latency to optogenetic CSD induction in VGAT-ChR2 slices in control (median = 14.0 seconds, mean ± SEM = 20.0 ± 2.3 seconds; *n =* 38), with VU0240551 (14.0, 21.0 ± 4.2 seconds, *n =* 12), with VU04663271 (13.0, 16.5 ± 3.5 seconds, *n =* 11), Isoguvacine (15.0, 19.0 ± 3.5 seconds, *n =* 11), or VU0463271 + Isoguvacine (12, 24 ± 9.1 seconds, *n =* 9) (Kruskal-Wallis test, *P =* 0.93). (**C**) CSD propagation speed in control (median = 3.42 mm/min, mean ± SEM = 3.38 ± 0.13 seconds; *n =* 38), with VU0240551 (2.89, 3.12 ± 0.26 mm/min, *n =* 12), with VU04663271 (3.28, 3.32 ± 0.18 mm/min, *n =* 15), Isoguvacine (3.12, 3.08 ± 0.25 mm/min, *n =* 11), or VU0463271 + Isoguvacine (3.00, 3.19 ± 0.16 mm/min, *n =* 9) (Kruskal-Wallis test, *P =* 0.93). (**D**) Success rate of optogenetic CSD in VGAT-ChR2 slices in control (100%, *n =* 39) with the Na^+^ channel blocker TTX 1 μM (0%; *n =* 13), with GABA-A (Gabazine 15 μM) and/or NMDA-AMPA-Kainate (CPP 10 μM, CNQX 20 μM) receptor antagonists (Gabazine 0%, *n =* 6; CCP+CNQX, 100%, *n =* 16; Gabazine+CCP+CNQX, 90%, *n =* 10), or the Ca^2+^ channel blocker Cd^2+^ (100 μM) to fully block synaptic release (100%, *n =* 10) (Fisher’s exact test, ****P = 4 × 10^–14^; Bonferroni-corrected posttest, ****P < 0.0001 for TTX). (**E**) Latency to optogenetic CSD induction in control (median = 14.0 seconds, mean ± SEM = 17.6 ± 2.5 seconds; *n =* 29), with Gabazine (10.0, 11.2 ± 3.0 seconds, *n =* 6), with CPP+CNQX (23.0, 25.8 ± 3.4 seconds, *n =* 16), CPP+CNQX+Gabazine (20.0, 26.6 ± 9.1 seconds, *n =* 9) or Cd^2+^ (36.6, 38.3 ± 7.6 seconds, *n =* 10) (Kruskal-Wallis test, ***P = 0.0098; Dunn’s post hoc test, *P < 0.05 for Cd^2+^). (**F**) CSD propagation speed in control (median = 3.45 mm/min, mean ± SEM = 3.57 ± 0.18 seconds; *n =* 30), with Gabazine (5.28, 5.08 ± 0.44 mm/min, *n =* 6), with CPP+CNQX (1.39, 1.51 ± 0.11 mm/min, *n =* 16), CPP+CNQX +Gabazine (2.66, 2.97 ± 0.21 mm/min, *n =* 9), or Cd^2+^ (0.9, 1.03 ± 0.13 mm/min, *n =* 10) (Kruskal-Wallis test, ****P < 0.0001; Dunn’s post hoc test, *P = 0.011 for CPP+CNQX and ****P < 0.0001 for Cd^2+^).

Next, in optogenetic experiments in which we used the K^+^ scavenger Kryptofix2.2.2 (2 mM) to chelate extracellular K^+^ (40) we observed a 5.6-fold reduction of optogenetic CSD success rate (Figure 6D). This confirmed the importance of extracellular K^+^ in the mechanism of CSD initiation, although chelation of basal extracellular K^+^ could also interfere with neuronal excitability. Even though our computational model points to extracellular K^+^ build-up directly induced by the spiking of GABAergic neurons, it has been shown that hyperactivity of GABAergic neurons can favor neuronal network excitation also by other mechanisms, including synaptic transmission–driven activation of neuronal-glial networks (41–44). Thus, we performed pharmacological experiments to disclose the detailed mechanism linking hyperactivity of GABAergic neurons to CSD initiation. In particular, the K^+^-Cl^–^ cotransporter KCC2 can induce postsynaptic K^+^ efflux and is involved in excitatory actions of GABAergic transmission leading to hyperexcitability (42). However, 2 different selective KCC2 inhibitors did not modify the success rate and dynamics (latency, propagation speed) of CSD induced by optogenetic stimulation, not even upon pretreatment of slices with the GABA-A receptor agonist isoguvacine that we used to increase KCC2 baseline activity (Figure 7, A–C). Further, we tested blockers of neuronal excitability or synaptic transmission. The Na^+^ channel/action potential blocker tetrodotoxin (TTX) completely suppressed CSD induction (Figure 7D), whereas the block of glutamate (Kainate-AMPA-NMDA) and/or GABA-A receptors (with CNQX-CPP and gabazine, respectively), or the application of the Ca^2+^ channel blocker Cd^2+^, which completely blocked synaptic transmission (Supplemental Figure 5), did not modify the success rate of CSD induction (Figure 7D). Latency to induction was instead longer with Cd^2+^ (Figure 7E). Notably, synaptic transmission was important for sustaining CSD propagation, in particular glutamatergic transmission, because in the presence of CNQX-CPP or Cd^2+^, the speed of CSD propagation was reduced (Figure 7F) and CSD often aborted, ending after an initial propagation around the initiation site (Supplemental Table). Gabazine induced a trend toward higher propagation speed (Figure 7F), which is consistent with the results that we obtained with *Scn1a*^+/-^ mice. These data highlight once more different mechanisms for initiation and propagation.

Altogether, these results demonstrate that GABAergic neuron hyperexcitability is sufficient for CSD initiation in the neocortex, that CSD is not directly triggered by ion flux through ChR2, and that synaptic transmission-driven mechanisms are not necessary, although they are implicated in propagation. Also, they suggest that the mechanism of initiation could involve spike-generated [K^+^]_out_ increase.

**Figure 8:**
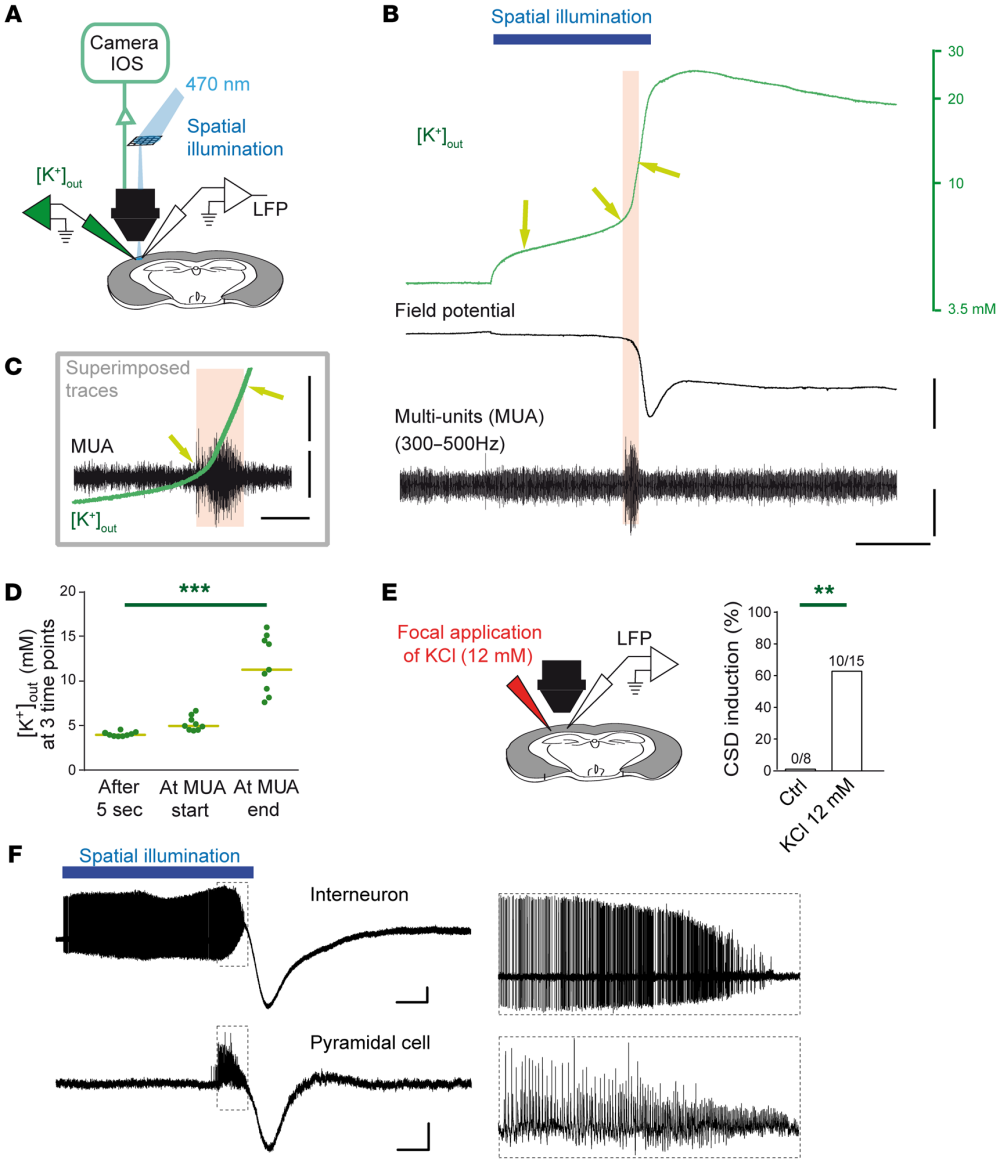
Spike-induced increase of [K^+^]_out_ is directly involved in CSD induction by spatial illumination. (**A**) Experimental setting for spatial 470 nm illumination used to specifically control the area of CSD induction, allowing [K**+**]_out_, LFP, and IOS recordings at the site of CSD initiation. See Supplemental Videos 6 and 7. (**B**) [K**+**]_out_ dynamics before and during CSD, correlated to the LFP and MUAs, which were paroxysmal at CSD initiation. Only the first component of the CSD is shown. (**C**) Enlargement and superposition of [K**+**]_out_ and MUA traces shown in **B**. (**D**) Quantifications of [K**+**]_out_ after the first 5 seconds of illumination, at the beginning of the paroxysmal MUA firing and at the end of the MUA firing (beginning of the depolarizing block) (arrows in **B **and **C**), *n =* 9 slices. Bars represent medians. Friedman test (*P <* 0.0001) and Dunn’s post hoc test (***P < 0.001). (**E**) Success rate of CSD induced by long-lasting puff of 12 mM KCl (dissolved in 125 mM NaCl), which corresponds to the [K**+**]_out_ at the beginning of the depolarizing block, compared with a control 137 mM NaCl solution (Ctrl; Fisher’s exact test, **P = 0.0027). Injection area: 0.75 ± 0.11 mm^2^ (*n =* 10 slices with successful CSD inductions). See Supplemental Video 8. (**F**) Representative simultaneous juxtacellular–loose patch recordings of a GABAergic interneuron (upper trace) and of a pyramidal neuron (bottom trace) at the site of initiation of CSD induced with spatial optogenetic illumination as in **A** (see Supplemental Video 9); the negative deflection is the LFP generated by the CSD and recorded by the juxtacellular electrode. Scale bars: 500 μV, 5 seconds. The right traces show the firing (1.5 Hz high pass filtered to remove the slow components) immediately before the CSD initiation (highlighted in the traces on the left with the dashed boxes). See text for *n* and statistics.

To disclose whether spike-generated [K^+^]_out_ increase was directly involved in CSD initiation, we first evaluated the [K^+^]_out_ dynamics during optogenetic illumination, measuring [K^+^]_out_ at the site of CSD initiation with K^+^-sensitive electrode recordings, together with LFP/multi-unit activity (MUA) recordings. In order to perform recordings at the site of initiation, which cannot be performed with classical methods of CSD induction, we used spatial optogenetic illumination, both continuous (Supplemental Videos 6 and 9) and discontinuous (100 ms, 5 Hz; Supplemental Video 7), to specifically control the site of CSD induction and allow recordings within the site (Figure 8, A and B). Both illumination methods induced CSD with features that were similar to those induced with the large field of illumination, although success rate was smaller (30%–35% versus about 80%). Our results show that [K^+^]_out_ slowly and progressively increased during illumination, reaching about 12 mM when CSD was ignited (identified as the end of the MUA; Figure 8, C and D and Supplemental Figure 6). Notably, this phase of slow increase was not observed in the CSD propagating in the cortical tissue outside the initiation site (Supplemental Figure 6). However, at CSD ignition, [K^+^]_out_ was similar to that observed at the site of initiation (Supplemental Figure 6). This suggests that at CSD initiation [K^+^]_out_ can progressively accumulate near neuronal membranes until CSD ignition threshold is reached. We confirmed this hypothesis by inducing CSD with focal applications of 12 mM KCl in a neocortical area that was comparable in size to that of spatial optogenetic illuminations (Figure 8E and Supplemental Video 8).

In order to study the dynamics of the firing of both GABAergic interneurons and pyramidal neurons in the site of CSD initiation, we performed juxtacellular-loose patch voltage recordings during CSD initiation (Figure 8F and Supplemental Video 9). Neurons were recorded in the initiation site during optogenetic triggering of CSD, using spatial illumination (34% success rate for CSD induction, 121 slices). Only cells that were located in the core of the CSD induction site were selected for analysis (*n =* 7 GABAergic neurons, *n =* 5 pyramidal neurons). Occasionally, pair recordings of GABAergic and pyramidal neurons were achieved (*n =* 2 pairs, Figure 8F). GABAergic neurons begun to fire early after the illumination (60.5 ± 11.3 seconds mean ± SEM, 52.3 seconds median, before the beginning of CSD initiation, *n =* 7) and fired at moderate frequency (on average 48.4 ± 6.8 Hz, mean ± SEM) till their firing frequency abruptly increased (to 349 ± 36 Hz, which was comparable to the maximal firing frequency observed in Figure 2C) a few seconds before CSD initiation. Thus, GABAergic neurons fired during the first (longer) phase at just about 15% of their maximal firing frequency. Differently than GABAergic neurons, pyramidal neurons fired later during the illumination, just for a few seconds before CSD initiation (2.9 ± 0.6 seconds mean ± SEM, median 3.5 seconds, *n* = 5; *P* = 0.006, Mann-Whitney test, comparing pyramidal and GABAergic neurons). Therefore, the firing of the GABAergic neurons at moderate frequency is implicated in the slow K^+^ build up observed at the site of initiation, whereas pyramidal neurons contribute later to the K^+^ build up, for a few seconds before CSD initiation, when the whole network is engaged.

### Overactivation of GABAergic neurons or Na_V_1.1 can initiate/facilitate CSD in vivo.

**Figure 9:**
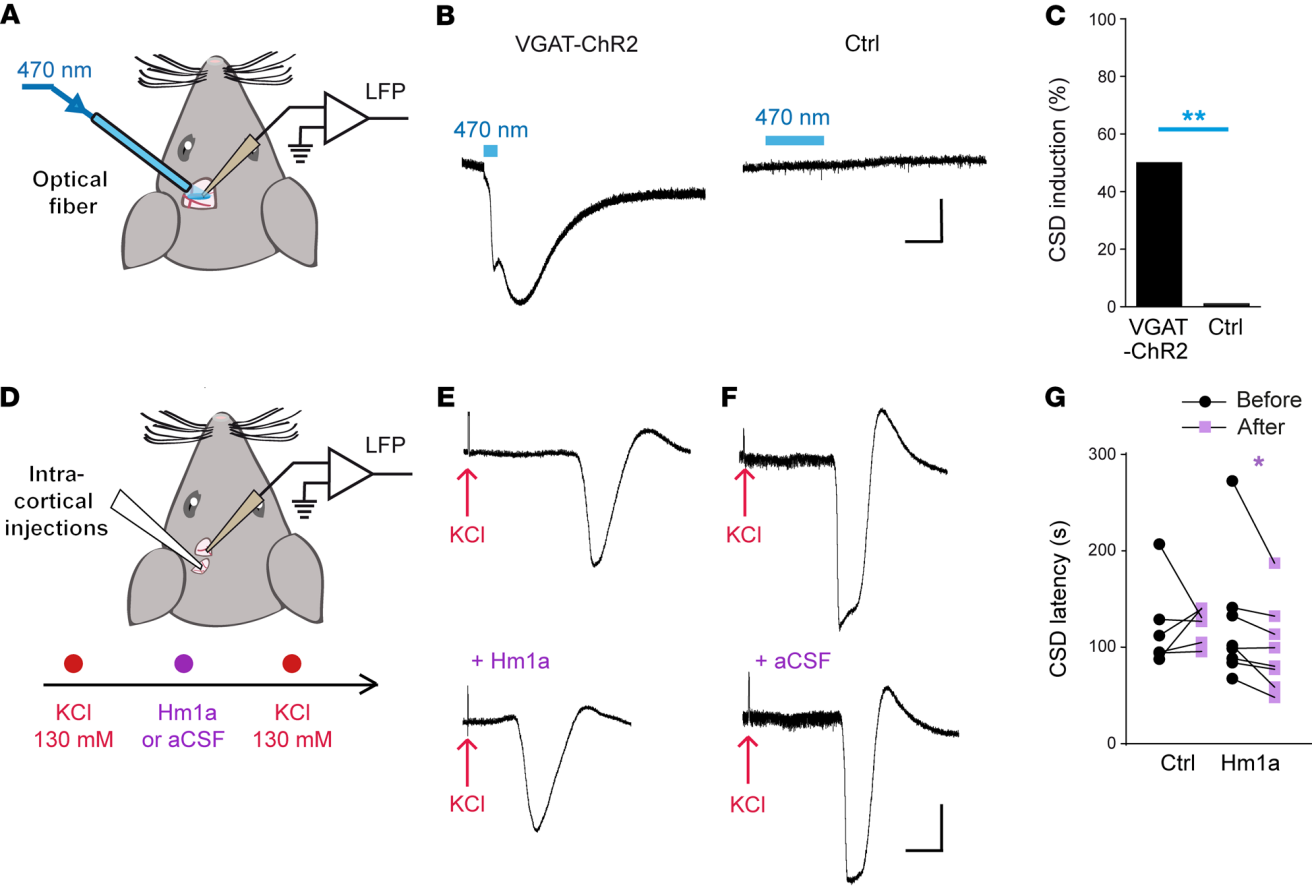
CSD induction in vivo. (**A**) Experimental design of optogenetic induction: blue light optogenetic stimulation (100 Hz trains of 0.8 ms pulses) was applied to the barrel cortex of anesthetized mice with an optical fiber through a craniotomy; DC field potential recordings were performed with a glass pipette (Ag/AgCl electrode). (**B**) Representative field potential traces of CSD in a VGAT-ChR2 mouse, whereas there was no response in a control WT mouse. (**C**) Proportion of optogenetic CSD induction in VGAT-ChR2 (5/10) and control mice (0/13, including WT, *n =* 5, VGAT.Cre, *n =* 4, and ChR2.lox, *n =* 4; Fisher’s exact test, **P = 0.0075). (**D**) Experimental design of Hm1a injections into the somatosensory cortex: CSD was induced by injecting 130 mM KCl with a 30-gauge needle and monitored by DC field potential recordings (1.3 mm more rostral), Hm1a or ACSF (negative control) were injected at the same location after CSD induction, and then a second CSD was induced by injecting again with 130 mM KCl. (**E**) Representative CSD traces obtained before (top) and after (bottom) the injection of 10 nM Hm1a. (**F**) Representative traces obtained before (top) and after (bottom) the injection of ACSF. (**G**) Comparison of CSD latency observed before and after injection of ACSF (control) or Hm1a 10 nM: 103 seconds (median), 121 ± 18 seconds (mean ± SEM) before ACSF; 129 seconds, 123 ± 8 seconds after ACSF (*n =* 6; Wilcoxon’s signed ranks test, *P =* 0.69); 100 seconds, 123 ± 23 seconds before Hm1a 10 nM; 90 seconds, 99 ± 16 seconds after Hm1a 10 nM (*n =* 8; Wilcoxon’s signed ranks test; *P = 0.016). Scale bars: 5 mV, 1 minute for all the panels.

We performed experiments to evaluate the effect of overactivation of GABAergic neurons or Na_V_1.1 in vivo, a condition in which blood circulation, long range connections, and neuromodulations are present. For optogenetic experiments, we illuminated the somatosensory cortex of VGAT-ChR2 mice with an optical fiber, monitoring CSD by LFP recordings (Figure 9, A–C). CSD was induced in 50% of VGAT-ChR2 mice and never in control littermates. Moreover, we tested the effect of acute injection of Hm1a into the somatosensory cortex (Figure 9D). We initially directly injected Hm1a (10 nM, *n =* 7, or 100 nM, *n =* 5), but we did not observe CSD with LFP recordings (1.3 mm rostral of the site of injection). In further experiments, we evaluated the effect of Hm1a on CSD triggered by injecting 130 mM KCl. Notably, 10 nM Hm1a induced a 20% reduction in the latency to CSD induction, whereas control injections with ACSF did not modify it (Figure 9, E–G). Moreover, injection of 100 nM Hm1a induced a 35% reduction in latency (117 seconds median, 108 ± 15 seconds, mean ± SEM, before Hm1a 100 nM; 64 seconds, 70 ± 14 seconds upon Hm1a injection; *n =* 7, Wilcoxon’s signed ranks test, *P =* 0.03). These data confirm the results obtained in brain slices, showing that overactivation of GABAergic neurons or Na_V_1.1 can lead to/facilitate CSD induction also in vivo.

## Discussion

We identified and characterized a mechanism of CSD initiation specific of the neocortex, showing in acute experimental models a causal relationship between Na_V_1.1 gain of function leading to initial hyperactivity of GABAergic neurons, progressive engagement of the whole neuronal network, and CSD ignition, driven by the progressive increase of [K^+^]_out_ at the initiation site and in which synaptic transmission is not necessary. This mechanism was supported by simulations obtained with a computational model. CSD was induced in the neocortex both by activation of Na_V_1.1 with the specific toxin Hm1a (34), which mimics gain-of-function FHM3 mutations and increases excitability of cortical GABAergic neurons, and by direct optogenetic activation of GABAergic neurons. This is consistent with the key role of Na_V_1.1 in GABAergic neuron excitability and with the effect of FHM3 mutations, which cause gain of function of Na_V_1.1 and can induce an increase of the persistent Na^+^ current similar to that observed with Hm1a (21–27, 29, 30, 37). Notably, it has been recently reported that Hm1a rescued the hypoexcitability of hippocampal GABAergic neurons and the severity of the epileptic phenotype in epileptic *Scn1a*^+/–^ knock-out mice, but it did not modify firing properties of WT hippocampal GABAergic neurons in brain slices (45). However, we found that application of Hm1a at a concentration at which it is specific for Na_V_1.1 can induce hyperexcitability of GABAergic neurons in neocortical slices from WT mice, in particular fast-spiking ones, whereas the firing properties of glutamatergic pyramidal neurons were not significantly modified, consistent with the different role of Na_V_1.1 in the 2 neuronal subtypes and with a specific role of GABAergic neurons in CSD initiation induced by Hm1a (see Supplementary Methods, “Brain Slices: Electrophysiological Recordings” for more information).

Additionally, we found that the loss of function of Na_V_1.1 in slices from VGAT-ChR2-*Scn1a*^+/–^ mice (17) inhibited the initiation of CSD by optogenetic stimulation, consistent with the involvement of Na_V_1.1 and of GABAergic neuron hyperexcitability in the mechanism of initiation. Importantly, different than what has been observed in some other epileptic models (46–49), this effect is not caused by the inhibition of CSD generation induced by the epileptic network, because threshold of CSD triggered by puffs of 130 mM KCl was not modified in VGAT-ChR2-*Scn1a*^+/–^ slices. Opposite to CSD inhibition, experiments in anesthetized mice have shown that Na_V_1.1 loss of function facilitates SD in the brainstem, which can cause postseizure sudden death (SUDEP) in *Scn1a*^+/–^mice because of block of cardiorespiratory pacemaking (7). However, this apparent discrepancy could be consistent with different mechanisms of initiation and propagation. In fact, contrary to CSD initiation, we observed a trend toward increased propagation velocity for optogenetic-induced CSD and higher propagation velocity for KCl-induced CSD in VGAT-ChR2-*Scn1a*^+/–^ slices. This effect could facilitate the generation/propagation of SD in the brainstem upon induction of epileptic discharges leading to postictal depression in the neocortex, as previously observed in *Scn1a*^+/–^ mice (7).

We also demonstrated that CSD initiation by direct GABAergic neuron hyperactivation is not dependent on synaptic transmission. In fact, the only manipulations able to inhibit CSD initiation were the generalized block of action potential (applying TTX) or the reduction of GABAergic neuron excitability (in brain slices from VGAT-ChR2-*Scn1a*^+/–^ mice), consistent with the simulations that we previously obtained (31). Differently than initiation, CSD propagation was inhibited by blocking Ca^2+^ channel–dependent synaptic release or glutamate receptors, but not by blocking GABA-A receptors. These results show that mechanisms linked to increased GABAergic synaptic transmission (41–44), possibly induced by GABAergic neuron hyperexcitability, are not involved in CSD. Moreover, glutamatergic transmission has an important role in propagation, but not in initiation when GABAergic neurons are overactivated. Overall, these results are consistent with different mechanisms of initiation and propagation. Notably, numerous studies have performed pharmacological investigations of mechanisms of CSD triggered with classic methods, revealing a complex picture with results that often are specific for different methods and for initiation versus propagation (2), consistent with different cellular/molecular mechanisms. Optogenetic experiments with spatial illumination (Figure 8 and Supplemental Figure 6) allowed us to investigate the properties of the site of initiation modeling the effect of FHM3 mutations. We have found that progressive K^+^ build-up induced by neuronal firing is a key factor at the site of CSD initiation by hyperactivation of GABAergic neurons, and chelation of extracellular K^+^ blocks CSD initiation. In our experiments, GABAergic neurons at the site of initiation initially fire at moderate frequency, sharply increasing their activity a few seconds before CSD initiation, when the whole network is engaged. This is a novel mechanism of CSD induction, different in comparison with that implicated in models of FHM1 and FHM2, in which it has been proposed that excessive glutamate release/accumulation is the major pathological dysfunction (13–15). Notably, a recent optogenetic CSD model of glutamatergic neuron hyperactivation probably mimics mechanisms at play in FHM1 and FHM2, including the necessity of NMDA receptor activation for CSD initiation (50).

Consistent with a different mechanism in comparison with FHM3, patients with FHM1 and FHM2 often show complex phenotypes that are more severe than those of FHM3 and include several neurologic/psychiatric comorbidities, including seizures that herald or are concomitant with hemiplegic migraine attacks, as well as peri-ictal death (3, 51). There are no patients with FHM3 with these complex phenotypes, and in the few cases in which seizures have been reported, they are always independent from migraine attacks and present in different developmental windows, as we recently reviewed (27). Supporting these clinical observations, a recent work reported that the knock-in mouse model of the FHM3 L263V Na_V_1.1 mutation shows spontaneous CSD events, but not seizures (33). Moreover, migraine or hemiplegia are not part of the phenotypes of epileptogenic Na_V_1.1 mutations that cause loss of function of the channel and hypoexcitability of GABAergic neurons (52–54), consistent with different pathologic mechanisms. Congruously, we have never observed ictal-like epileptiform activities in our experiments, although it has been shown that ictal-like activities generated by application of convulsants in brain slices could be enhanced/induced (43, 55) or, depending on the brain region, inhibited (56) by the optogenetic activation of GABAergic neurons. This probably reflects the requirement of a network that already generates epileptic activities for induction/modulation of these activities by the activation of GABAergic neurons. Nevertheless, it is interesting to note that, in some of those studies, different mechanisms of onset (some requiring activation of GABAergic neurons, others activation of glutamatergic neurons) have been identified for epileptiform activities that appear phenomenologically similar (43). This could be the case, as we have already highlighted, also for network activities that lead to CSD initiation, which could be specific for different types of migraine, in particular for FHM3 compared with FHM1 and FHM2.

In our experiments, latencies to CSD induced by activation of GABAergic neurons were of a few tens of seconds. In most of the classical experimental models, CSD is induced with strong stimuli (e.g., injections of KCl in the tens of millimolars to molar range, the latter well beyond pathophysiological limits; ref. 2), which often result in shorter latencies most likely because they do not reproduce the complete dynamic process of CSD onset at the initiation site, mimicking conditions that are those of the generalized depolarization phase. There is no information about activities of single neurons leading to CSD induction in migraine patients. As in other episodic and paroxysmal disorders (57), pathologic dysfunctions of migraine are probably controlled by homeostatic mechanisms in the period between attacks, and different factors (e.g., hormonal/neuromodulatory changes or increase of incoming neuronal signals from the periphery) may affect neuronal excitability and activities of cortical networks, triggering CSD induction and migraine attacks. It can be hypothesized that, in the restricted volume of cortex in which CSD is initially initiated, these factors could weaken homeostatic controls and hyperexcitable GABAergic neurons could be hyperactivated (at moderate firing frequency) for tens of seconds (even intermittently, as in Supplemental Video 7), similar to our acute model. Interestingly, neurons can show an early and long-lasting increase of activity in other episodic neurological disorders, as observed with single unit recordings in epileptic foci of patients (58), in which increased neuronal activity can begin minutes before the attack.

The accompanying paper by Auffenberg et al. (37) provides results that complement our work. In fact, the authors studied the pathogenic mechanisms of the human FHM3 L1649Q *SCN1A* mutation in a knock-in mouse model, mechanisms that we previously studied in expression systems (25). They demonstrated that, in an animal model, L1649Q induces Na_V_1.1 gain of function (in particular because of slowed and incomplete inactivation, an effect that is similar to that of Hm1a), which causes enhanced firing of GABAergic interneurons, in particular PV+ fast-spiking ones (consistent with our experiments showing that their overactivation can be sufficient to trigger CSD), without modifications of pyramidal neuron firing, leading in vivo to facilitation of CSD induction. Importantly, our work provides additional evidence because it discloses detailed mechanisms of CSD initiation that cannot be studied with standard models, and shows that acute hyperactivation of Na_V_1.1/GABAergic neurons is sufficient to induce CSD in the normal (nonpathologic) neocortex. Future studies for investigating even better detailed pathological mechanisms of FHM3 using the now-available knock-in mouse models are warranted. However, in comparison with chronic models (i.e., genetic), our acute models allowed us to study mechanisms and dynamics of CSD initiation and demonstrate that other possible pathological modifications (e.g., remodeling of gene expression) are not necessary.

In conclusion, we have disclosed a mechanism of CSD initiation specific of the neocortex in which GABAergic neurons play a key role, involved in the pathological mechanism of FHM3 mutations. Migraine etiology is multifactorial, with probably numerous different mechanisms (3). This mechanism of CSD initiation may be implicated in other types of migraine and possibly in other pathologies in which SDs are involved (e.g., stroke, traumatic brain injury, and subarachnoid hemorrhage; ref. 1), because it could be associated not only to Na_V_1.1 mutations, but also to other dysfunctions that lead to GABAergic neuron hyperactivity.

## Methods

See Supplementary Methods for additional details.

### Animal care and mouse lines.

All efforts were made to minimize the number of animals used and their suffering. Mice were housed as a group (5 mice per cage, or 1 male and 2 females per cage for breeding) on a 12 hour light/dark cycle, with water and food ad libidum. Mouse lines were obtained from Jackson Laboratory (JAX), except GAD67-GFP knock-in mice (59), which were obtained from Yuchio Yanagawa (Gunma University, Maebashi, Japan).

To have specific expression of ChR2-H134R/tdtomato in GABAergic neurons, most of the experiments were performed with male and female double hemizygous transgenic mice VGAT-hChR2(H134R)/tdtomato (VGAT-ChR2) and control littermates (F1 generation). They were obtained by mating hemizygous females loxP-STOP-loxP-hChR2(H134R)-tdTomato (Ai27D, B6.Cg-Gt(ROSA)26Sortm27.1(CAG-COP4*H134R/tdTomato)Hze/J; JAX catalog 012567; ref. 60) with transgenic males (to avoid off-target Cre expression in the female germline) hemizygous for the Viaat-Cre transgene (Cre recombinase expression driven by the vesicular GABA transporter [VGAT] promoter) (B6.FVB-Tg(Slc32a1-Cre)2.1Hzo/FrkJ; JAX catalog 017535), line 2.1 as previously described (36). To avoid germline transmission of recombined floxed alleles (www.jax.org/strain/017535), we never used VGAT-ChR2 F2 offspring. VGAT-Cre mice have been previously used for obtaining specific expression of floxed alleles in GABAergic neurons (36, 61–64). To evaluate the effect of a reduction of Na_V_1.1 expression in interneurons, we crossed double hemizygous VGAT-ChR2 mice with heterozygous Na_V_1.1 knock-out mice (*Scn1a*^+/–^; ref. 17). To express ChR2 selectively in PV+ neurons, we crossed hemizygous loxP-STOP-loxP-hChR2(H134R)-tdtomato female mice with homozygous Pvalb-IRES-Cre (JAX catalog 017320) male mice. Moreover, for immunohistochemistry, we used mice expressing a floxed td-tomato transgene (Ai9, B6;129S6-Gt(ROSA)26Sor^tm9(CAG-tdTomato)Hze^/J; JAX catalog 007905) as a cell filling reporter line, because it was difficult to identify hChR2(H134R)/tdtomato–expressing cells with the plasma membrane fluorescence of the tagged ChR2 in VGAT-ChR2 mice.

All mouse lines were in the C57BL/6J background (>10 generations, Charles River), except *Scn1a*^+/–^ mice, which were in a mixed background (C57BL/6J-CD1 85%:15%).

Offspring was genotyped either by PCR following the standard JAX protocols and using our standard protocol for *Scn1a*^+/–^ knock-out mice (65) or, for mice with cell filling fluorescent protein, controlling the fluorescence of newborn mice with a Dual Fluorescent Protein Flashlight (NightSea). We used mice of both sexes, 4 to 6 weeks old for ex vivo experiments and 4 to 8 weeks old for in vivo experiments.

### Preparation of brain slices, electrophysiological recordings, and imaging in slices.

Brain slices were prepared and patch-clamp recordings performed as previously described (19, 35). Local field potential recordings and recordings with K^+^ selective electrodes were performed as previously described (19, 35, 65, 66). IOS imaging was performed as previously described (35). 

### Optogenetic illumination of brain slices.

Activation of ChR2 was obtained by illuminating brain slices through the 4× objective with blue light generated using a white light source (Intensilight, Nikon) and appropriate filters. Spatial illumination was performed with a digital micromirror device–based (DMD-based) patterned photostimulator (Polygon 400, Mightex). We used either continuous illumination or trains of illumination (5 Hz, duty cycle 50%). 

### Induction of CSD by application of KCl in brain slices.

Brief puffs of KCl (130 mM) and Fastgreen (0.1%, Sigma-Aldrich; to visualize the injection area) were applied in the superficial cortical layers (layers 2–3) with a glass micropipette (2–4 MΩ) connected to an air pressure injector, as previously described (35).

### Processing and analyses of IOS images.

CSD waves obtained by intrinsic optical imaging were processed and analyzed with ImageJ-Fiji as previously described (35). 

### Immunohistochemistry.

Brains were fixed with paraformaldehyde 4%. Experiments were performed on 40 μm coronal sections. Image acquisitions were obtained with a confocal laser-scanning microscope (FV10i, Olympus).

### In vivo experiments.

Mice were deeply anesthetized with ketamine/xylazine (100 mg/kg and 5 mg/kg, respectively) and DC field potentials were recorded with a glass pipette filled with ACSF inserted into the barrel cortex through a craniotomy. Optogenetic stimulations were applied on the surface of the cortex with a 400 μm diameter optical fiber, connected to a 470 nm LED light source (100 Hz trains of 0.8 ms pulses); Hm1A or control ACSF was injected with a 30-gauge needle. Mice were sacrificed at the end of the recordings by cervical dislocation.

### Patch-clamp recordings in cell lines.

Plasmids were propagated, cells transfected, and whole-cell, patch-clamp recordings performed and analyzed as previously described (25, 67). 

### Pharmacological agents and chemicals.

CNQX, VU0240551, and VU0463271 were from Tocris Bioscience; CPP and TTX-citrate were from Alomone Labs; Kryptofix2.2.2 was from Thermo Fisher Scientific. Synthetic Hm1a was purchased from Smartox S.A.S. All other chemicals were purchased from Sigma-Aldrich. Induction of CSD was tested after 15 minutes of perfusion of the slices with the drug or drugs.

### Computational model.

The model of 2 coupled neurons, GABAergic and pyramidal, was based on the one we developed previously (31). In the present study, we refined the model introducing the modifications described in the Supplementary Methods, and performed the numerical simulations with the software XPPAUT (http://www.math.pitt.edu/~bard/xpp/xpp.html). The code is available at http://modeldb.yale.edu/267157

### Statistics.

For experiments with mice, we used data pooled from at least 3 animals per condition (including negative controls) to ensure reproducibility of results. Mice were used after genotyping and littermates were negative controls. In mice expressing fluorescent proteins, systematic tests using a flashlight were performed before the experiment to confirm the genotyping results. Statistical tests were performed with Origin 8 (OriginLab) and R. Fisher’s exact test followed by a pairwise test adjusted with Bonferroni correction was performed for the analysis of contingency tables. Two-tailed nonparametric Mann–Whitney *U* test*,* Kruskal-Wallis test, and Friedman 1-way ANOVA were performed because groups were not large enough to accurately verify normality and equality of variance. Dunn’s nonparametric comparison and Bonferroni correction (comparisons with the control group) were used for post hoc tests when appropriate. The 1-sample Wilcoxon’s signed ranks nonparametric 1-sided test was used to evaluate the effect of Hm1a on Na^+^ currents in cell lines (fold increase of I_NaP_ larger than 0) and the paired Wilcoxon’s signed ranks test was used for effect of Hm1a on firing in brain slices. Differences were considered significant at *P* less than 0.05.

### Study approval.

Experiments were carried out according to the ARRIVE guidelines and the European directive 2010/63/UE and approved by institutional and ethical committees (approvals PEA 362/APAFIS 7848-2016112114249820v3 for France, 711/2016-PR for Italy).

## Author contributions

OC, SZ, PS, LP, AL, MA, IL, and SC performed experiments and collected data. OC, SZ, PS, LP, AL, FD, IL, SC, and MM analyzed and interpreted the data. LL, MK, MD, and MM implemented the computational model and interpreted the simulations. OC, SZ, IL, SC, and MM designed the experiments. OC, SZ, PS, LL, AL, SC, and MM prepared the figures. OC and MM wrote the manuscript. OC and SZ equally contributed to the work; OC is listed as first co–first author because she also shared leadership with MM in the experimental part of the work.

## Supplementary Material

Supplemental data

Supplemental Video 1

Supplemental Video 2

Supplemental Video 3

Supplemental Video 4

Supplemental Video 5

Supplemental Video 6

Supplemental Video 7

Supplemental Video 8

Supplemental Video 9

## Version 1 09/07/2021

In-Press Preview

## Version 2 11/01/2021

Electronic publication
