# Supplementary material for: Initiation of migraine-related cortical spreading depolarization by hyperactivity of GABAergic neurons and NaV1.1 channels
Source: J Clin Invest. 2021 Nov 1;131(21):e142203. doi: 10.1172/JCI142203 (PMC8553565; doi:10.1172/JCI142203)
Supplement: Supplemental data [file jci-131-142203-s214.pdf]

Supplementary Material

Initiation of migraine-related Cortical Spreading Depolarization by hyperactivity of GABAergic neurons and $Na_V1.1$ channels.

Oana Chever[1,2,^#], Sarah Zerimech[1,2,#], Paolo Scalmani[3], Louisiane Lemaire[4], Lara Pizzamiglio[1,2], Alexandre Loucif[1,2], Marion Ayrault[1,2], Martin Krupa[5], Mathieu Desroches[4], Fabrice Duprat[1,2,6], Isabelle Lena[1,2], Sandrine Cestèle[1,2] and Massimo Mantegazza[1,2,6*]

1 Université Côte d'Azur, Institute of Molecular and Cellular Pharmacology (IPMC), Valbonne-Sophia Antipolis, France.
2 CNRS UMR7275, Institute of Molecular and Cellular Pharmacology (IPMC), Valbonne-Sophia Antipolis, France.
3 U.O. VII Clinical and Experimental Epileptology, Foundation IRCCS Neurological Institute Carlo Besta, Milan, Italy.
4 Inria Sophia Antipolis Méditerranée, MathNeuro project-team, Valbonne Sophia Antipolis, France.
5 Université Côte d'Azur, Laboratoire Jean-Alexandre Dieudonné, Nice, France.
6 Inserm, Valbonne-Sophia Antipolis, France.

# Co-first author

^ Present address: Normandie University, UNIROUEN, INSERM U1239, DC2N, 76000 Rouen, France

Corresponding author:
Massimo Mantegazza: mantegazza@ipmc.cnrs.fr
Institute of Molecular and Cellular Pharmacology (IPMC), CNRS UMR 7275 and Université Côte d'Azur, 660 Route des Lucioles, 06560 Valbonne-Sophia Antipolis, France. Tel. +33 (0)493953425 FAX +33 (0)493957708

Graphical abstract- Legend

Focal GABAergic neurons' hyperactivity (long lasting firing at moderate frequency) (1) caused by pathologic dysfunctions (e.g. $Na_V1.1$ gain-of-function) can lead to spiking-induced accumulation of extracellular K^+ (2), which drives the network to hyperexcitability (3) and eventually induces depolarization block and CSD initiation, independently from synaptic transmission. This mechanism can be associated not only to gain-of-function $Na_V1.1$ mutations, but possibly also to other dysfunctions that induce GABAergic neurons' hyperactivity. As in all episodic disorders, homeostatic mechanisms can control dysfunctions in the period between the attacks, but triggering factors (e.g. hormonal/neuromodulatory changes or increase of incoming neuronal signals from the periphery) may focally affect neuronal excitability and activities of cortical networks, leading to long lasting GABAergic neurons' hyperexcitability and CSD induction.

Supplementary methods

Brain slices: preparation, electrophysiological recordings and imaging.

Brain slices were prepared as previously described (1, 2). Briefly, mice were killed by decapitation under isoflurane anesthesia, the brain was quickly removed and placed in ice-cold artificial cerebrospinal fluid (ACSF), which contained (in mM): 125 NaCl, 2.5 KCl, 2 $CaCl_2$, 1 $MgCl_2$, 1.25 NaH_2PO_4, 25 $NaHCO_3$ and 25 glucose, saturated with 95 % O_2 - 5 % CO_2. Acute coronal slices 400µm thick (300µm for spatial illumination and patch-clamp experiments) were prepared with a vibratome (HM650V, MicroM, Germany) in ice-cold ACSF. Slices were then stored in a submerged chamber with ACSF at 34°C at least one hour before the beginning of the recordings. Selective brain expression of the ChR2(H134R)/tdTomato transgene and thus absence of germline recombination of the floxed allele was systematically confirmed by visual inspection of slices and of other tissues of the mouse using the NightSea Flashlight (USA). Inductions of CSD started > 10min after placing an individual slice in the recording chamber (RC-26GLP; Warner Instruments, USA), which was perfused with "recording ACSF" (rACSF) at 34°C, containing (in mM): 125 NaCl, 3.5 KCl, 1 $CaCl_2$, 0.5 $MgCl_2$, 1.25 NaH_2PO_4, 25 $NaHCO_3$ and 25 glucose, saturated with 95% O_2 - 5% CO_2. This rACSF has been already used for studying CSD (3), and our control experiments did not show statistically significant differences in CSD properties compared to standard ACSF (Supplementary Fig.7) Slices were visualized using infra-red differential interference contrast (DIC) microscopy (Nikon Eclipse FN1, Japan) equipped with a CCD camera (CoolSnap ES2, Photometrics, USA), filter cubes for visualization of fluorescent proteins (Semrock, USA) and a filter for optogenetic 475nm blue light illumination (FF02-475/50; Semrock, USA). Acquisitions of large fields of view were obtained with a 0.35X camera adapter lens. Electrophysiological signals were recorded with a Multiclamp 700B amplifier (CV-7B headstage), a Digidata 1440A acquisition board and pClamp 10.3 software (Molecular Devices, USA). DC extracellular field potential recordings were performed using borosilicate glass micropipettes (~0.5MΩ) filled with rACSF. Whole-cell patch-clamp recordings were performed using borosilicate glass pipettes of 3–5MΩ resistance containing (in mM): 120 K-gluconate, 15 KCl, 2 $MgCl_2$, 0.2 EGTA, 10 HEPES, 20 P-Creatine, Na_2-0.2 GTP, 2 Na_2-ATP, 0.1

leupeptin, adjusted to pH 7.25 with KOH. Whole-cell access resistance (10-25MΩ) was monitored and cells showing instable access resistance (> 20%) were discarded. Recordings were started 5min after obtaining the whole-cell configuration. Juxtacellular-loose patch recordings were performed with the same pipettes used for whole cell experiments, but filled with rACSF, and action potentials were recorded in the I=0 mode.

For pyramidal neurons, we have recorded randomly from these neurons in neocortical layer II-III. $Na_V1.1$ is expressed also in pyramidal neurons, even if at much lower level than in most GABAergic neurons, but there is not yet consensus about which type of excitatory neuron expresses it. In fact, although some works have reported expression of $Na_V1.1$ in discrete subpopulations of neocortical pyramidal neurons and no expression in hippocampal pyramidal neurons (4, 5), numerous others have instead found low level of $Na_V1.1$ expression in most pyramidal neurons of both hippocampus and neocortex, although loss of function of $Na_V1.1$ does not in general modify firing properties of these neurons (6-11). Moreover, in a BAC transgenic mouse expressing exogenous $Na_V1.1$ (12), which is a model similar to that used by (5), Na^+ currents generated by the exogenous $Na_V1.1$ were observed in all the neurons (both GABAergic and pyramidal) randomly selected upon dissociation from the neocortex (12), although the amount of current recorded in the different neurons was dependent on the specific transgenic line. Thus the lack of effect of Hm1a in pyramidal neurons in our whole-cell recordings of Figure 2 shouldn't be caused by the random selection of pyramidal neurons not expressing $Na_V1.1$. Additionally, the neocortical specificity of CSD induction that we have observed cannot anyway be explained by the selective expression of $Na_V1.1$ in cortical excitatory neurons, because we obtained neocortical-specific CSD induction also with optogenetic stimulations, which are selective for GABAergic neurons.

Ion sensitive electrodes were built with glass pipettes (tip diameter ~ 2µm) as previously described (13). Briefly, they were first cleaned with absolute ethanol, then pre-treated with dimethylchlorosilane vapors (Sigma-Aldrich, USA), dried at 100°C for 2h, and finally the tip was backfilled with the K^+ ionophore I-cocktail B (Fluka, Sigma-Aldrich, USA) using a 28G microfil (WPI, USA). The calibration was

done for each microelectrode using solutions of different K^+ concentrations (2.5, 3.5, 35, and 90mM: KCl added to the ACSF), and electrodes were selected according to the slope of their response (a minimum of 50mV/decade increase in K^+ concentration). To minimize the leak current of the headstage in ion sensitive recordings, we used Rf = 5GΩ and compensated the residual leak current: we zeroed the pipette offset observed with a 10MΩ load, we then switched to a 10GΩ load and we re-zeroed the offset observed in this condition (which is mainly due to the residual leak current) with the current injection circuitry of the amplifier (holding current); the estimated leak current (equal and opposite to the holding current applied) was 0.73pA at 25°C and stable over time. For accurate evaluation of $[K^+]_{out}$ dynamics, the field potential signal was subtracted from the K^+-sensitive recording (the ion sensitive and the extracellular potential pipettes were placed at <100µm of distance from each other). Electrophysiological signals were filtered at 10kHz and sampled at 25kHz. Multi-unit activities (MUA) were obtained band-pass filtering off-line the LFP trace at 300-500Hz with pClamp (single pole RC filters). Intrinsic optical signal (IOS: near-infrared light transmittance) (14) was monitored acquiring images with the CoolSnap ES2 CCD camera controlled with micromanager (15) at 1 image/s (unless otherwise indicated); note that videos in supplementary material are accelerated (5 to 50 image/s). Analyses and signal/image processing were performed using pClamp 10.3, Origin 8 (Origin Lab, USA) and ImageJ-Fiji (16).

Optogenetic illumination of brain slices.

Activation of ChR2 was obtained illuminating brain slices through the 4x objective. A white light source (130W mercury lamp, Intensilight, Nikon, Japan) was connected to the epi-illumination port of the microscope with a light guide containing a 420nm UV blocker filter (series 2000, Lumatec, Germany); the white light was filtered with a 475/50 filter (Semrock) placed in the optical path and delivered to the objective with a FF685-Di02 dichroic beamsplitter (Semrock). The area of illumination was 38.5mm^2. The blue light power density measured with a power meter (Ophir Photonics, USA) was 2.8mW/mm^2 at the slice surface (photodetector placed at the level of the slice). For spatial illumination

experiments, the light guide was connected to a digital micromirror device (DMD)-based patterned photostimulator (Polygon 400, Mightex, Canada), which was connected to the microscope with a custom adapter and a NI-FLT6 Epi-fluorescence Cube Turret equipped with a FF685-Di02 dichroic beamsplitter and a 475/50 filter. The area of the spatial illumination was between 0.34 and 1.1mm^2. ON/OFF of the illumination was controlled with the Intensilight shutter or with the Polygon 400 software. We mainly used continuous illumination, but trains of illumination have been also successfully tested (with spatial illumination: 5Hz, duty cycle 50%; see Video 7). The infrared filter of the microscope, besides for patch-clamp experiments, was used in most experiments to eliminate the blue light of the optogenetic illumination from the acquired images and to obtain near-infrared IOS.

Induction of CSD in brain slices by application of KCl.

Cortical spreading depression was induced by brief puffs of KCl (130mM) with a glass micropipette (2-4MΩ) connected to an air pressure injector (holding pressure: < 1PSI, injection pressure: 7 to 10PSI; PV820 Pneumatic Picopump, WPI, USA) (1). The field potential recording pipette was placed at least 500µm away from the CSD induction area. For CSD induction with a solution containing 12mM KCl (12mM KCl, 125mM NaCl, negative control solution was 137mM NaCl saline solution), long local perfusions of KCl were performed with a glass pipette of 0.5MΩ (3PSI) until CSD induction. For both brief puffs and long local perfusions, Fastgreen (0.1%, SIGMA-Aldrich, USA) was added to the pipette solutions to visualize the injection area. Application of the control NaCl solutions with Fastgreen never ignited CSD.

Processing and analyses of IOS images.

Image analysis with ImageJ-Fiji was used for identifying the CSD wave by intrinsic optical imaging, as in (1). Image processing was performed to evaluate latency of CSD initiation and to quantify CSD propagation speed with a custom made macro (available at https://www.ipmc.cnrs.fr/~duprat/scripts/imagej.htm): to eliminate the background and isolate the

wave from the raw image, a representative image acquired before the 470nm illumination was subtracted from the others and then contrast was enhanced. To determine the speed of the propagating wave, successive line plots of the wave front from processed images (every 2s) were drawn manually and the spatial distances between them were measured by means of the peak finder ImageJ plugin. For each slice, speed was estimated on a minimum of 4 time points (8s). For few experiments, the quality of the images was not sufficient for a reliable quantification of CSD propagation speed, and they were not included in the analysis for the quantification of the speed. Latencies of CSD initiation were quantified off-line using processed images. Only CSDs that initiated in the visual field of the camera were used for latency analysis.

For the evaluation of the percentage of CSD induction**,** individual slices were exposed to 470nm illumination for 90s maximum. When the illumination did not induce CSD within this time limit, we tested that the slice could generate CSD by injecting a puff of 130mM KCl solution as described above. The percentage of optogenetically induced CSD was quantified considering as non-successful optogenetic inductions only experiments in which the slice could generate CSD with a subsequent application of KCl. The same protocol was used to determine the success rate of spontaneous CSD induction following bath application of Hm1a toxin: individual slices from VGAT-ChR2 mice were perfused with rACSF or with Hm1a (10nM) dissolved in rACSF for 15min. If no CSD occurred in this time window, then optogenetic CSD induction was tested. The percentage of CSD triggered following Hm1a was quantified considering as non-successful inductions the experiments in which the slice could generate CSD with a subsequent 470nm illumination. Threshold of CSD induced by short puffs of 130mM KCl was quantified as in (1), evaluating the area of injection by drawing the limits of the dark zone observed upon injection of the fast-green containing solution and obtaining with ImageJ-Fiji the value of the enclosed area, which was increased by repeating the injections (inter-injection interval 1min) until CSD was ignited, which defined the CSD threshold. "Aborted CSD" refers to CSD that rapidly decelerate after initiation and stop propagating within <800µm from the initiation site.

Immunohistochemistry, confocal acquisitions, and cell count.

Mice were anesthetized (pentobarbital, 40mg/kg, IP) and intracardially perfused with cold PBS (Sigma-Aldrich, USA) and paraformaldehyde 4% (Electron Microscopy Sciences, USA) prepared in PBS. Brains were removed and post-fixed overnight. 40 µm-coronal sections were performed with a microtome (HM650V, Microm, Germany). Before immunohistochemistry processing, floating sections were permeabilized and immunoblocked with Triton X-100 (0.1%, Sigma-Aldrich, USA), NGS (10%, Normal Goat serum, S-1000, Vector lab, USA), BSA (0.5%, Bovine serum albumin, Amresco, USA) diluted in PBS, at RT for 30min. Then, slices were incubated overnight at 4°C with primary antibody, and after several washes at RT, with the appropriated secondary antibody. Antibodies were diluted in PBS containing Triton X-100 (0.1%), NGS (1%), BSA (0.5%). Slices were mounted on glass slides using Fluoroprep (Biomérieux, France) or Mowiol (Sigma Aldrich, USA) as mounting media. All antibodies used in the study are commercially available and have been validated in previous published studies, as reported by the suppliers.

Primary antibodies used: anti-NeuN (1/1000) mouse monoclonal antibody (MAB377, Merck Millipore, USA), anti-CamKII (1/100) mouse monoclonal antibody (6G9 clone, MA1-048, ThermoFisher Scientific, USA), anti-S100 (1/1000) rabbit polyclonal antibody (Z0311, Dako Corp., USA). Fluorescent dye-conjugated secondary antibodies used in appropriate combinations: Goat-anti mouse or anti-rabbit antibodies (1/1000: Alexa 647, A21236 and A21245, Invitrogen, USA).

Immunofluorescence acquisitions were performed using a confocal laser-scanning microscope (FV10i, Olympus, Japan), with a 60x objective (image format: 1024x1024). Mosaics of ~1.5mm x 1.5mm were performed with 635nm and 559nm laser lines. Quantification of fluorescent cells and co-localisation of fluorescent markers were performed with ImageJ-Fiji and the cell counter plugin, using 200µm-width regions of interest (ROI) drawn perpendicularly to the cortical surface and covering all the cortical layers. 4-5 ROI were used for each mouse and the grand average was then performed with 3 mice.

Patch-clamp recordings in cell-lines.

Plasmids, cell culture and transfections. The cDNAs of the $Na_V1.1$ Na^+ channel (GenBank sequence NM_006920.4) and of the $Na_V1.2$ Na^+ channel (GenBank sequence NM_021007) were obtained from Jeff Clare (Glaxo-SmithKline, UK) and subcloned into the pCDM8 vector to reduce rearrangements, as already described (17, 18). The plasmids were propagated in MC1061/P3 *E. Coli* (Invitrogen-ThermoFisher, USA) grown at 28°C for > 48h, and the entire coding sequence was sequenced after each propagation to rule out the presence of unwanted spurious mutations or rearrangements. $Na_V1.1$ or $Na_V1.2$ were co-expressed with a reporter vector expressing YFG (pEYFP-N1) in tsA-201 cells (authenticated cells purchased from ECACC-Sigma: cell line 96121229) transiently transfected by using the $CaPO_4$ method, as previously described (17, 18); tsA-201 cells were cultured in modified Dulbecco's medium and Hams-F12 mix supplemented with 10% fetal bovine serum (Invitrogen-ThermoFisher, USA). $Na_v1.6$ Na^+ channel (GenBank sequence NM_014191.2) was recorded from a stably expressing HEK293 cell line (8) (obtained under a MTA from Jeff Clare, Glaxo-SmithKline, UK), which were cultured in modified Dulbecco's medium supplemented with 10% foetal bovine serum (Invitrogen-ThermoFisher, USA). Cell cultures were tested for mycoplasma contamination (LookOut kit, Sigma-Aldrich).

Whole-cell recordings and analysis. Na^+ currents were recorded with the whole-cell configuration of the patch-clamp technique (24-48h after transfection for $Na_V1.1$ and $Na_V1.2$) as previously described (17, 18). The recordings were performed at room temperature (22-25°C) using a Multiclamp 700A amplifier and pClamp 10.2 software (Axon Instruments/Molecular Devices). Signals were filtered at 10kHz and sampled at 100kHz. Electrode capacitance and series resistance were carefully compensated throughout the experiment. Pipette resistance was between 1.5 and 2.5MΩ; maximum accepted voltage-clamp error was 2.5mV. The remaining transient and leakage currents were eliminated online using a P/4 subtraction paradigm. The extracellular recording solution contained (in mM): 150 NaCl, 10 HEPES, 2 KCl, 1.5 $CaCl_2$ and 1 $MgCl_2$ (pH 7.4 with NaOH); the internal pipette solution contained (in mM): 105 CsF, 35 NaCl, 10 EGTA, 10 HEPES (pH 7.4 with CsOH).

***In vivo* experiments.**

Male or female mice (4-8 weeks of age) were deeply anesthetized with ketamine/xylazine (100mg/kg and 5mg/kg, respectively) and placed in a Faraday cage-shielded stereotaxic frame (Narishige Instruments, Tokyo, Japan). Supplemental doses of anesthesia were applied on appearance of withdrawal reflex in response to limb pinching. The body temperature was maintained at 37-37.5°C (rectal probe) with a heating pad connected to a temperature controller (TCAT2DF, Physitemp Instruments, USA); the stability of the respiratory activity was monitored with a piezoelectric transducer (MLT1010 Pulse Transducer, AD Instruments, UK) and that of the heart rate with ECG recordings.

For optogenetic experiments, a craniotomy was performed (-2mm antero-posterior, 3.5mm lateral with respect to bregma), the dura was carefully removed, and mineral oil (Sigma-Aldrich) was applied on the cortex to prevent cortical surface drying. A glass pipette filled with 0.9% NaCl (1-2µm tip diameter, Ag/AgCl electrode) was lowed into the barrel cortex for recording DC field potentials. The placement of the pipette in the barrel cortex was confirmed recording the spiking activity induced by vibrissae stimulations. A 400µm diameter optical fiber, connected to a 470nm LED light source (Optoflash, Cairn research, UK; 30mW/mm^2 at the optical fiber tip) was placed on the surface of the cortex, in the area of the recording pipette. The photostimulations consisted of 100Hz trains of 0.8ms pulses, delivered till a CSD was triggered; after 100s the stimulation was considered unsuccessful. Note that trains of illumination were able to induce CSD also in brain slices (Video 7).

For evaluating the effect of Hm1a on KCl-induced CSD, two burr holes were drilled at the level of the somatosensory cortex (coordinates relative to Bregma: AP= - 1.7 or - 0.4mm, respectively; lateral= ± 3.0mm, according to the mouse brain Atlas of Paxinos and Watson (19)), one for injections (KCl or Hm1a toxin), the second, more rostral, for extracellular DC recordings. Injections were performed using a 30-gauge needle connected via a polyethylene tubing to a 1ml syringe controlled by a pump (AL-1000, World Precision Instruments, Sarasota, USA). In initial experiments, we injected Hm1a toxin

10nM or 100nM (in ACSF, 2.5µl at a rate of 0.33µl/min). In further experiments, we induced CSD by unilateral injection of 130mM KCl at a rate of 1µl/min into the barrel cortex (-1.5mm from the skull surface). KCl was applied (for 5min maximum) until a CSD was observed. Hm1a toxin (10nM or 100nM in ACSF) or ACSF were injected (2.5µl) at the same stereotaxic coordinates at a rate of 0.33µl/min and the needle was left *in situ* for an additional 5min to prevent back flow before removal. Two KCl-induced CSD were generated at 15min intervals before and after injection of Hm1a or ACSF. Latencies between the beginning of cortical KCl injection and the onset of CSD were measured. For each hemisphere, the mean of latencies obtained for the two CSD before Hm1a or ACSF injection was compared to that observed after the injection.

DC field potentials were recorded with a glass micropipette filled with ACSF and a differential extracellular amplifier (EX4-400, Dagan, USA), and acquired with a Digidata 1440A and pClamp software (Molecular Devices, USA). A reference Ag/AgCl electrode was placed on the cortical surface. Anesthetized mice were sacrificed at the end of the recordings by cervical dislocation.

Computational model

We revised the model developed in (20) better modeling the dynamics of ionic concentrations, which is an essential feature in CSD. In fact, in (20), the reversal potentials of the conductances of the GABAergic neuron did not depend on the ion concentrations, and only some of the transmembrane currents of the pyramidal neuron had an effect on ion concentrations and reversal potentials. Here, we took into account the effect of each transmembrane current I_{ion} on the intracellular ion concentration $[ion]_{in}$ of the corresponding neuron and on the extracellular ion concentration $[ion]_{out}$. For each neuron, we have

$$C\,\frac{dv}{dt} = -\sum_{\mathrm{ion}\,\epsilon\,S} I_{\mathrm{ion}},$$

$$\frac{d[\mathrm{ion}]_{in}}{dt} = -\frac{\gamma}{z_{\mathrm{ion}}} I_{\mathrm{ion}} \text{ for ion } \in S = \{\mathrm{Na}^+, \mathrm{K}^+, \mathrm{Cl}^-\}.$$

C is the membrane capacitance per area unit, v the membrane potential, t the time, z_{ion} the valence

and γ a conversion factor. We replaced the Wang-Buzsáki model of the interneuron that we used in (20) with a more recent model, which better models features of fast-spiking cortical interneurons (21). Moreover, we modified the GABAergic neuron leak current implementing Na^+ (0.012 mS cm^{-2}) and K^+ (0.05 mS cm^{-2}) leak conductances, so that the GABAergic neuron does not spike in the absence of external input and its resting potential is in the physiological range.

Compared to (20), we set the ratio of the GABAergic neuron/pyramidal neuron volume to 2/3, and, to include the impact of excitatory synaptic currents I_{GLU} on the dynamics of ion concentrations, we separated its Na^+ $I_{Na,GLU}$ and K^+ $I_{K,GLU}$ components, assuming an equal permeability of the glutamatergic receptors to both ions:

$$I_{GLU} = I_{Na,GLU} + I_{K,GLU} = \frac{g_{GLU}}{2} s_e(v - E_{Na}) + \frac{g_{GLU}}{2} s_e(v - E_K),$$

where g_{GLU} is the maximal conductance, s_e the pyramidal neuron synaptic variable, and E_{Na} and E_K the reversal potentials. We modeled external inputs (drives) to the neurons using constant glutamatergic currents (which did not depend on the glutamate released by the synapses of the modeled neurons), whose amplitude was set by varying their conductances: $g_{D,e}$ for the pyramidal neuron and $g_{D,i}$ for the GABAergic neuron. We included the activity of the Na/K ATPase in the dynamics of ion concentrations for both neurons, not only for the pyramidal one as in (20). We replaced the expression describing the dependence of the pump current I_{pump} on the intracellular Na^+ and extracellular potassium concentrations with a more realistic one developed by (22), which is based on experimental data:

$$I_{pump} = \rho_{pump}(v) \left(\frac{[Na^+]_{in}}{[Na^+]_{in} + K_{Na}} \right)^3 \left(\frac{[K^+]_{out}}{[K^+]_{out} + K_K} \right)^2.$$

We introduced a voltage dependence of the pump as in (23), to prevent the membrane potential from reaching excessively negative values when recovering from a depolarization block:

$$\rho_{pump}(v) = \rho_{pump,-70} \frac{f(v)}{f(-70)},$$

$$f(v) = \frac{1 + tanh\,(0.39\frac{v}{26.64} + 1.28)}{2}.$$

We used the half activation concentration values K_{Na} and K_K from (24) and a maximal pump rate at −70 mV $\rho_{pump,-70} = 30\ \mu A\ cm^{-2}$. We also modified a number of additional minor aspects. First, we increased the maximal conductance of the Na^+ leak current of the pyramidal neuron to 0.015 mS cm^{-2}, so that its resting membrane potential is not overly negative. We also set the maximal conductance of the calcium-activated potassium current of the pyramidal neuron to 1 mS cm^{-2}, the maximal conductance of the inhibitory synaptic current to 0.1 mS cm^{-2} and the rate of extracellular potassium diffusion to 0.00004 ms^{-1}. For consistency, in the equation of the intracellular calcium concentration of the pyramidal neuron, we used the same factor γ as for the other ions to convert current density to rate of change in intracellular concentration.

We implemented the effect of Hm1a and of FHM3 mutations by replacing part of fast inactivating Na^+ current of the GABAergic neuron $I_{Na,FI}$ with a persistent Na^+ current $I_{Na,P}$, keeping the sum of their maximal conductances $g_{Na,FI} + g_{Na,P}$ constant; 1% of persistent current was considered as the control physiologic condition. The equation modeling the persistent Na^+ current was similar to the one for the fast inactivating one, with the difference that there was no inactivation (we set the inactivation variable h to 1) and that the voltage dependence of the activation variable m_∞ was shifted to more negative potentials by 10mV (25, 26):

$$I_{Na,FI} = g_{Na,FI}\, m_\infty^3\,(v)\, h(v)\,(v - E_{Na}), I_{Na,P} \ = \ g_{NaP}\, m_\infty^3\,(v + 10)\,(v - E_{Na}).$$

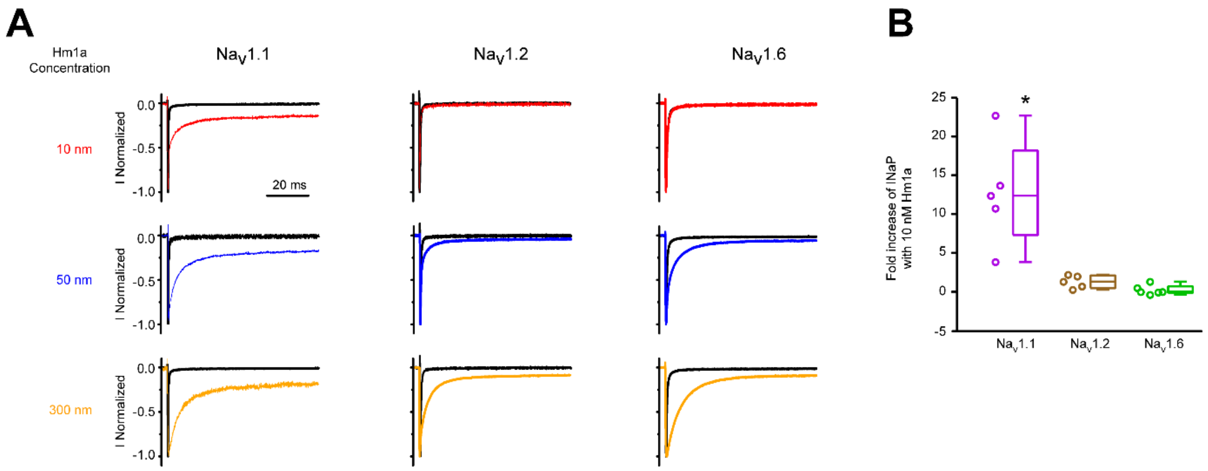

Supplementary Figure S1. Test of the selectivity of our synthetic Hm1a peptide on Na^+ channel isoforms expressed in the central nervous system: $Na_v1.1$, $Na_v1.2$ and $Na_v1.6$. A. Representative whole-cell patch-clamp recordings of Na^+ currents evoked with 100ms depolarizing steps to -10mV from a holding potential of -100mV in tsA-201 cells expressing $Na_v1.1$, $Na_v1.2$, or $Na_v1.6$ in control condition or in the presence of 10, 50 or 300nM Hm1a. Apparent EC50 (efficacy concentration at 50% of maximum effect) was 3nM for $Na_v1.1$ (n=5 cells), 53nM for $Na_v1.2$ (n=5 cells) and 41nM for $Na_v1.6$ (n=6 cells). Note the selective enhancing effect of 10nM Hma1 on $Na_v1.1$ compared to $Na_v1.2$ and $Na_v1.6$. At higher concentrations, Hma1 loses its selectivity on $Na_v1.1$. **B.** Quantification of the effect of 10nM Hm1a displayed as fold increase of I_{NaP}, showing that Hm1a is selective towards $Na_v1.1$ over $Na_v1.2$ and $Na_v1.6$ isoforms. Whiskers box-chart plots represent median, minimum and maximum. One-sided one sample Wilcoxon Signed Rank Test, * p=0.029.

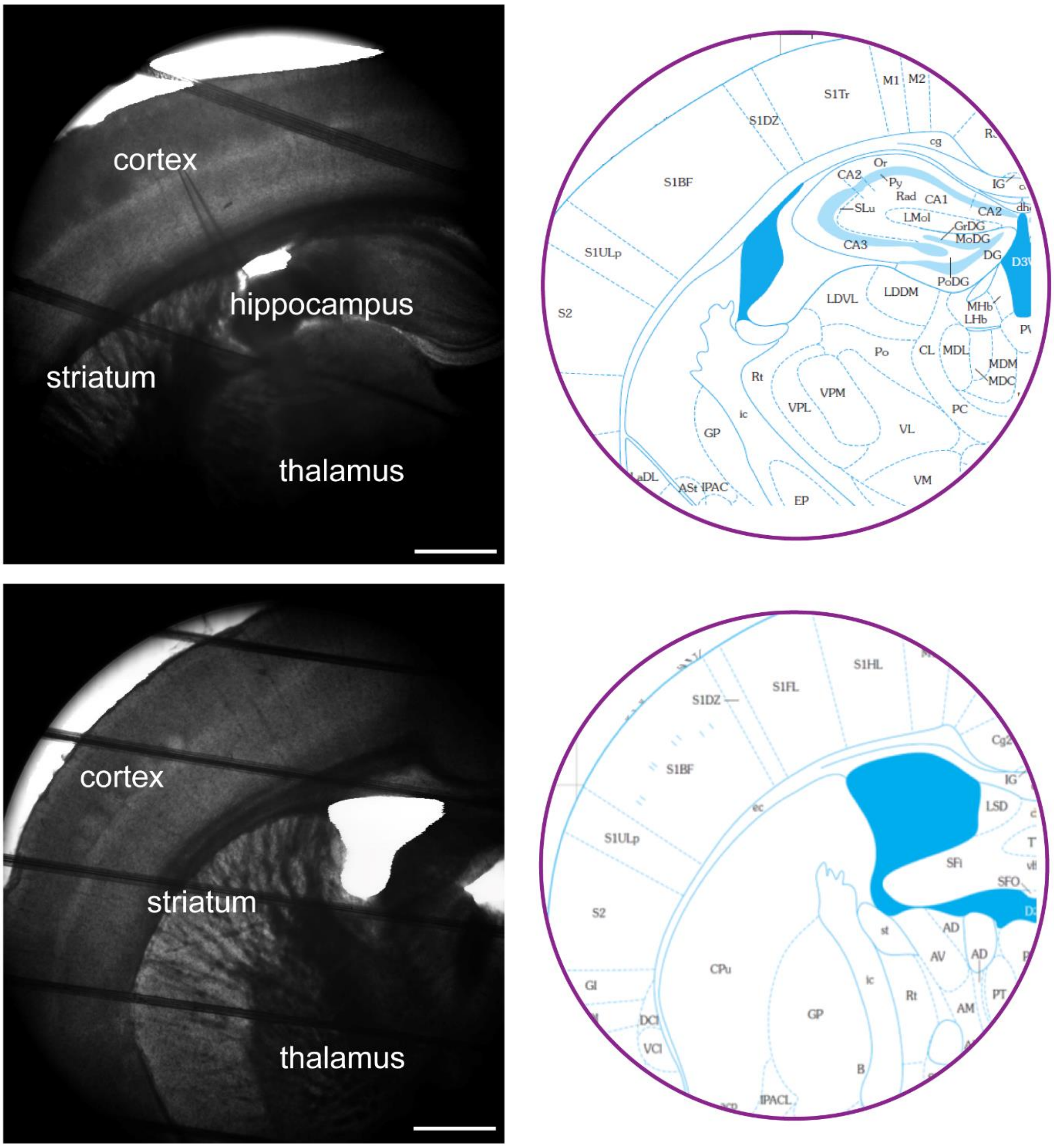

Supplementary Figure S2. Imaged areas and anatomical structures in representative slices from experiments in which was triggered with Hm1a. Left; same panels displayed in Fig. 1D and 1E. **Right;** corresponding imaged areas illustrated on the diagrams of Paxinos and Franklin's Mouse Brain in Stereotaxic Coordinates 3rd (top panel Bregma 1.46, Interaural 2.34mm, bottom panel Bregma -0.82, Interaural 2.98mm) for displaying in more detail the anatomical structures included. AD: anterodorsal thalamic nucleus. AM: anteromedial thalamic nucleus. ASt: amygdalostriatal transition area. AV: anteroventral thalamic nucleus. B: basal nucleus (Meynert). CA1: field CA1 of the hippocampus. CA2: field CA2 of the hippocampus. CA3: field CA3 of the hippocampus. Cg: cingulum. CL: centrolateral thalamic nucleus. CPu: caudate putamen (striatum). D3V: dorsal 3rd ventricle. Dhc: dorsal hippocampal commissure. Ec: external capsule. EP: entopeduncular nucleus. GI: granular insular cortex. GrDG: granule cell layer of the dentate gyrus. Ic: internal capsule. IG: indusium griseum. IPAC: interstitial nucleus of the posterior limb of the ant. LaDL: lateral amygdaloid nucleus, dorsolateral part. LDDM: laterodorsal thalamic nucleus, dorsomedial part. LDVL: laterodorsal thalamic nucleus, ventrolateral part. LHb: lateral habenular nucleus. LMol: lacunosum moleculare layer of the hippocampus. LSD: lateral septal nucleus, dorsal part. M1: primary motor cortex. M2: secondary motor cortex. MDC:

mediodorsal thalamic nucleus, central part. MDL: mediodorsal thalamic nucleus, lateral part. MDM: mediodorsal thalamic nucleus, medial part. MHb: medial habenular nucleus. MoDG: molecular layer of the dentate gyrus. Or: oriens layer of the hippocampus. PC: paracentral thalamic nucleus. Po: posterior thalamic nuclear group. PoDG: polymorph layer of the dentate gyrus. PT: paratenial thalamic nucleus. Py: pyramidal cell layer of the hippocampus. Rad: radiatum layer of the hippocampus. Rt: reticular nucleus (prethalamus). S1BF: primary somatosensory cortex, barrel field. S1DZ: primary somatosensory cortex, dysgranular zone. S1FL: primary somatosensory cortex, forelimb region. S1HL: primary somatosensory cortex, hindlimb region. S1ULp: primary somatosensory cortex, upper lip region. S2: secondary somatosensory cortex. SFi: septofimbrial nucleus. SFO: subfornical organ. SLu: stratum lucidum of the hippocampus. VL: ventrolateral thalamic nucleus. VM: ventromedial thalamic nucleus. VPL: ventral posterolateral thalamic nucleus. VPM: ventral posteromedial thalamic nucleus.

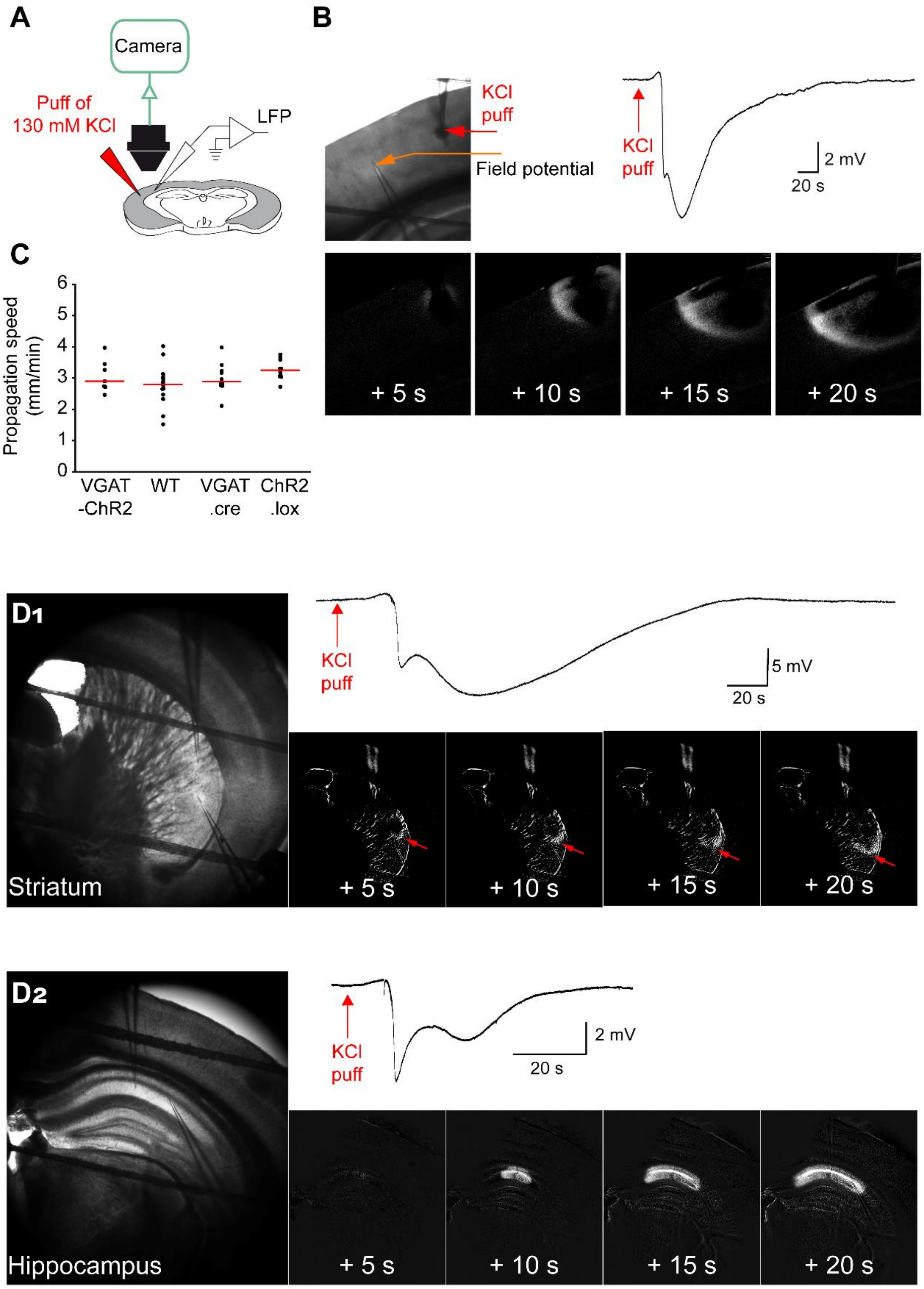

Supplementary Figure S3. CSD is readily triggered by focal puffs of KCl 130mM in neocortex, striatum and hippocampus. A. Experimental design of KCl-induced CSD, which was triggered by a focal puff of 130mM KCl applied using a picopump (10PSI, 500ms). Fastgreen (0.1%) was added to the KCl solution to visualize the effective KCl injection and quantify its area (mean=0.030 ± 0.005mm^2, n=10 slices). **B.** Representative CSD induced in the neocortex and revealed by both a negative DC shift in the LFP and the propagating wave observed with intrinsic optical signal (IOS) imaging. The four lower panels show representative IOS images of CSD at different time points (image processing of raw images to better

highlight the CSD wave, see methods). Scale bars 250µm. See Video 2. **C.** CSD was induced by 130mMKCl and had similar properties in different mouse lines: the dot plot displays the propagation speed in slices from VGAT-ChR2 mice (line that we used for optogenetic experiments, in which CSD has been induced with 130mM KCl after unsuccessful optogenetic illuminations; n=7 slices), WT littermate mice (n=14), VGAT.cre mice (n=10) and ChR2.lox mice (n=10) (Kruskall-Wallis test, p=0.32); for the last three conditions the slices are those presented in Fig.3D (success rate plot). Bars correspond to medians. Pooling all the data, the CSD ignited by the KCl puff propagated in the cortical tissue at the speed of 3.01 ± 0.08mm/min (mean ± SEM, n=41 slices). **D_1**. Representative CSD induced in the dorsal striatum by a puff of 130mM KCl as in B for the neocortex (n=7, 100% success rate); transmitted light image of the imaged area (left panel) and time series of processed images (right lower panels); the right upper panel shows the LFP recording; the CSD wave front is indicated by the red arrows. Scale bars 500µm. See Video 3. **D_2**. Representative CSD induced in the hippocampus by a puff of 130mM KCl as in B for the neocortex (n=8; 100% success rate); transmitted light image of the imaged area (left panel) and time series of processed images (right lower panels); the right upper panel shows the LFP recording. Scale bars: 500µm. See Video 4.

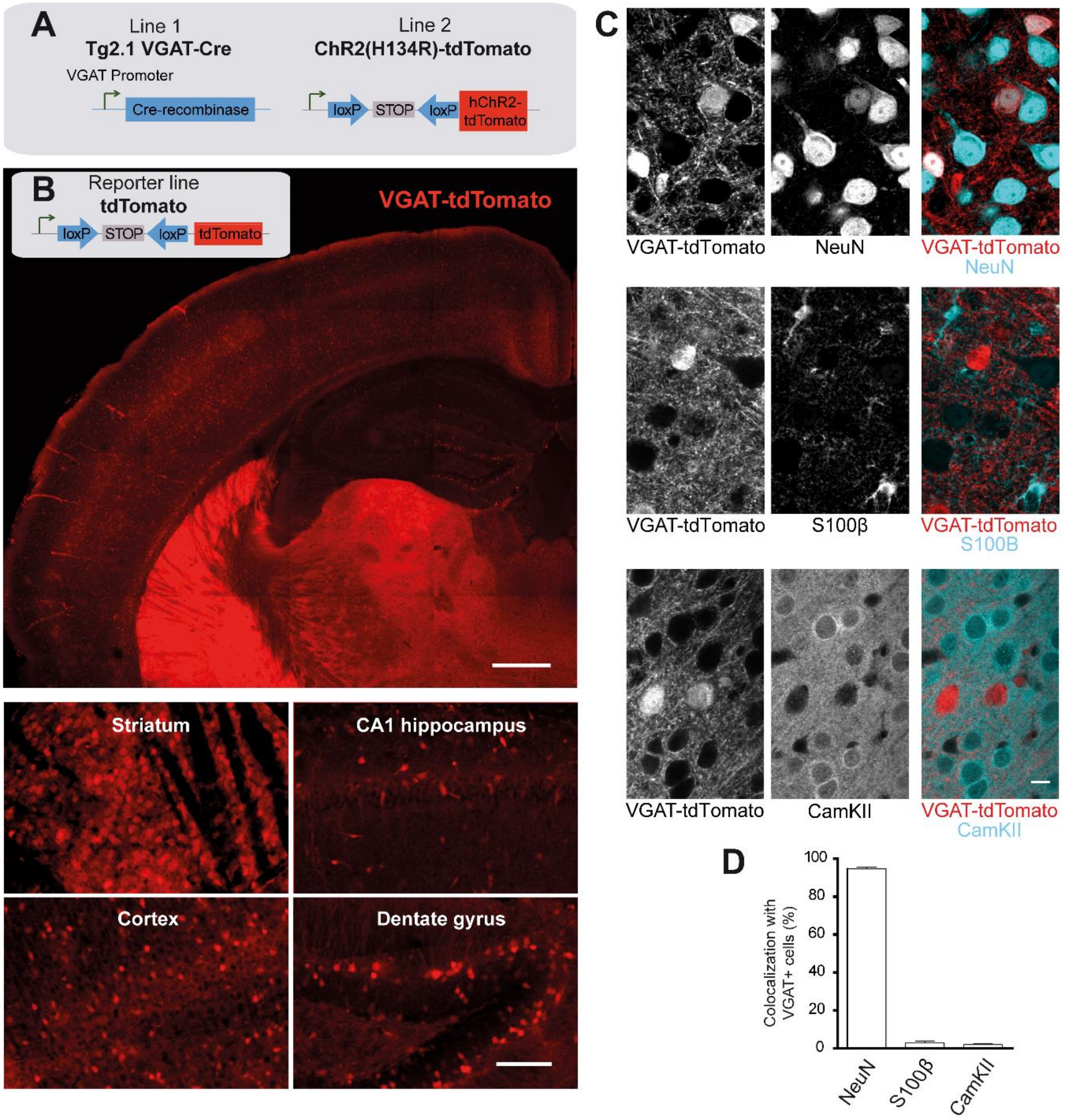

Supplementary Figure S4. ChR2 is expressed selectively in GABAergic neurons and in numerous brain areas of VGAT-ChR2 mice. A. Mouse lines crossed for generating VGAT-ChR2 mice used for optogenetic experiments. **B.** Extended mosaic confocal image of a representative coronal brain slice from the mouse reporter line VGAT-tdTomato, in which VGAT-Cre+ cells (i.e. GABAergic neurons) express the cytoplasmatic tdTomato fluorescent protein (red), showing that the VGAT promoter drives expression in numerous brain areas including neocortex, hippocampus, striatum and the thalamus neuropil. Scale bar: 500µm. The lower panels display magnified areas from the upper panel, with single cell resolution. Scale bar: 100µm **C.** Representative confocal images of neocortical immunostainings with a neuronal marker (NeuN), an astrocytic marker (S100β) and a marker of excitatory neurons (CamKIIa) in tissue from VGAT-tdTomato mice. Scale bar: 10µm **D.** Quantifications of NeuN+ cells (95 ± 1%, grand average of 3 mice), S100β+ cells (3.0 ± 0.9%, grand average of 3 mice), and CamKII+ cells (2.0 ± 0.5%, grand average of 3 mice) among neocortical VGAT-tdTomato cells, confirming that ChR2 in VGAT-ChR2 mice is not expressed in excitatory neurons and astrocytes. We have not used VGAT-ChR2 mice in these experiments because the fluorescence of tdTomato-tagged ChR2 is mainly limited to the plasma membrane, and thus difficult to identify for an accurate quantification at the cellular level.

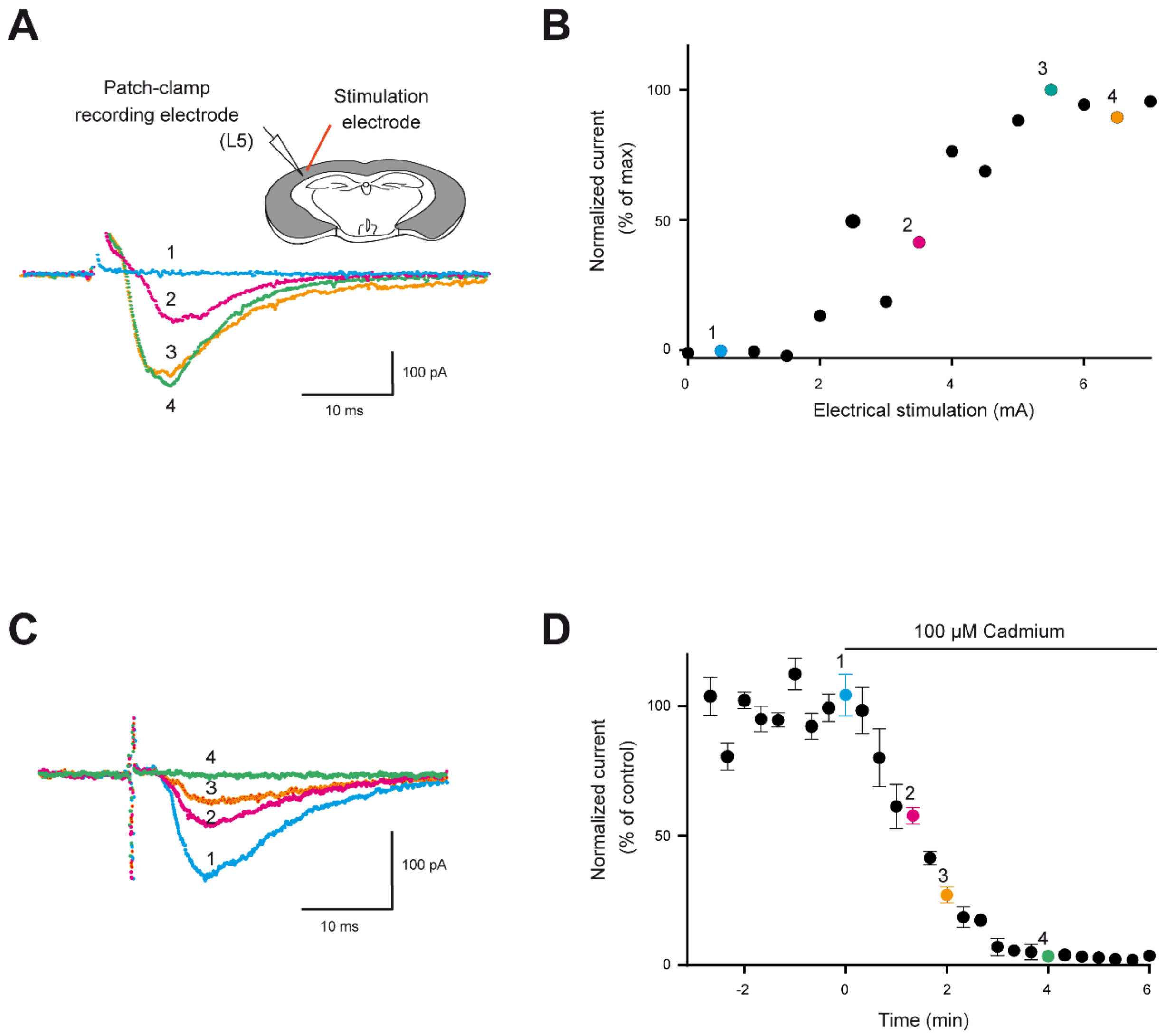

Supplementary Figure S5. A. Representative post-synaptic currents (EPSCs) recorded from a Layer 5 pyramidal neuron (holding potential −60mV), evoked by Layer 2/3 single stimulations (0.05Hz) with incremental intensities. Schematic representation of the experimental design is depicted in the top right inset. **B.** Representative input-output curve from this cell, allowing the determination of the intensity evoking the maximal EPSC amplitude. **C.** Representative EPSCs before and during bath application of the Ca^{2+} channel blocker Cd^{2+} (100µM), with stimulation that evoked the maximal EPSC responses. **D.** Time course of the blocking effect of Cd^{2+} on normalized maximal EPSC amplitudes, consistent with complete block of synaptic transmission. Each point represents the mean of normalized currents ± SEM (n=5 cells). Same colors were used in representative traces and pooled data. One-sided paired Wilcoxon Signed Rank Test (before and after 4min of Cd^{2+} perfusion, p=0.031).

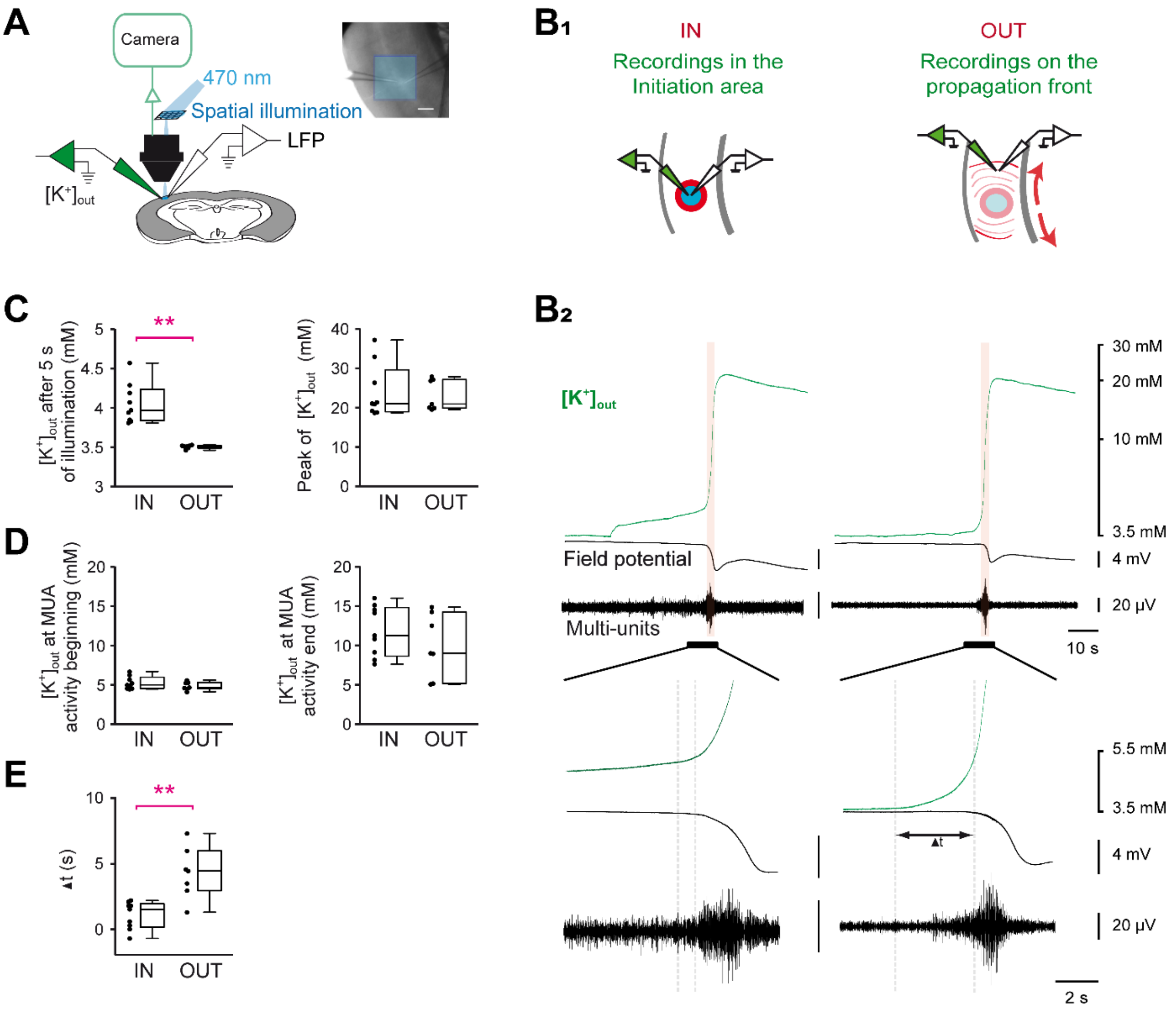

Supplementary Figure S6. Comparison of $[K^+]_{out}$ within and outside the CSD initiation area. A-B_1. Experimental design to compare dynamics of $[K^+]_{out}$ of the CSD ignited using spatial illumination. Scale bar: 250µm. **B_2.** Representative traces of $[K^+]_{out}$ dynamics before and during CSD initiation in both areas, with associated local field potential recordings and multi-unit activities (MUA). The vertical orange area highlights the period of paroxysmal MUA, which corresponds to the transient intense neuronal firing that precedes the long lasting depolarization in CSD. The bottom panel highlights, with an enlarged time scale, the time lags (Δt, dotted lines) between the increase in $[K^+]_{out}$ (at the foot of the abrupt increase observed during the CSD) and the beginning of the negative DC deflection of the field potential. **C-D.** Dot and box-chart plots (Whiskers represent minimum and maximum) of $[K^+]_{out}$ inside (IN; n=9) and outside (OUT; n=7) the CSD initiation zone after 5 seconds of illumination (C left panel, Mann-Whitney test, *** p=0.001), at the maximal peak amplitude (C right panel, Mann-Whitney test p=0.76), at the beginning of paroxysmal MUA (D left panel, Mann-Whitney test p=0.53) and at the end of paroxysmal MUA (D right panel, Mann-Witney test, p=0.31). **E.** Dot and box-chart plot of time lags between the beginning of the sharp $[K^+]_{out}$ increase and the beginning of the negative DC shift of local field potentials, as shown in B_2 (Mann-Whitney test, ** p=0.0059). Note the slow increase of $[K^+]_{out}$ in the initiation area during optogenetic illumination until CSD triggering, in contrast to the sharp $[K^+]_{out}$ increase recorded during CSD propagating outside the initiation area. CSDs recorded "inside" and "outside" the illumination area were triggered at the same $[K^+]_{out}$ concentration (~12mM; end of MUA activities) (D). Recordings in the initiation area have been already presented in Figure 8.

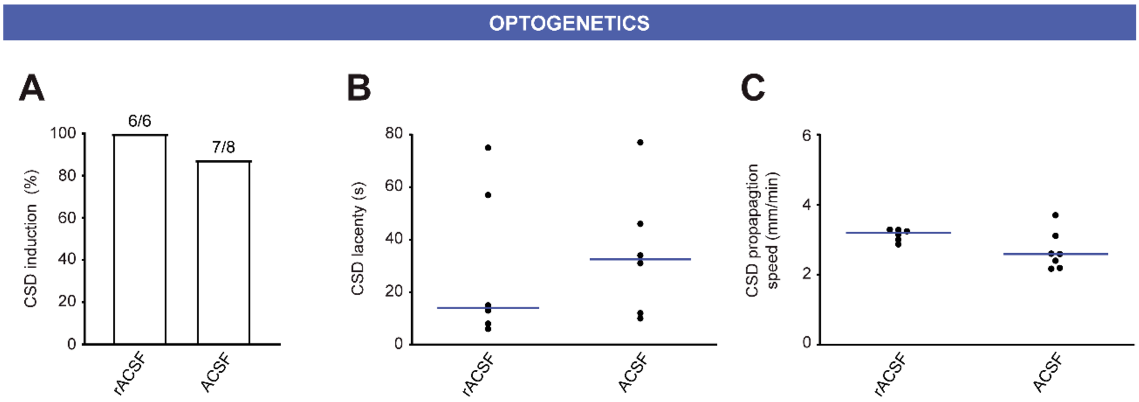

Supplementary Figure S7. Optogenetic CSD induction with rACSF and ACSF. Properties of optogenetic CSD induced in slices from VGAT-ChR2 mice were not different in rACSF compared to ACSF. In this series of experiments, success rate (**A**) was 100% in rACSF (6 successes in 6 slices) and 87.5% in ACSF (7 successes in 8 slices) (Fisher's exact test, p=1), latency of induction (**B**) had median of 14s and mean±SEM of 29±12 s with rACSF and 32.5s, 35±10s with ACSF (Mann Whitney test, p=0.59; one CSD in ACSF has been removed because the initiation site was outside the imaged area), CSD propagation speed (**C**) had median of 3.2mm/min and mean±SEM of 3.14±0.07mm/min with rACSF and 2.6mm/min 2.7±0.2mm/min with ACSF (Mann Whitney test, p=0.07).

Proportion of aborted CSD (%)

	Ctrl	GABAZINE	CPP CNQX	CPP CNQX GABAZINE	CADMIUM
n	0/30 (0%)	0/6 (0%)	4/16 (25%)	0/9 (0%)	7/10 (70%)
Statistics	Fisher's exact test p=3.6^{e-06}				
Pairwise test adjusted with Bonferroni correction		NS	=0.0111 (*)	NS	<0.0001 (***)

Supplementary Table. Quantification of the number of CSD induced by 470nm illumination in brain slices that spontaneously ended after initiation within 800µm from the initiation site (reported as aborted CSD). "n" refers to the number of slices.

Legends to Supplementary Videos

All the videos are played by Windows Media Player Version 12.0.18362.900 on Windows 10

Video 1. Application of the selective $Na_V1.1$ enhancer Hm1a specifically induces CSD in the neocortex. Upper panels: Intrinsic Optical Signal (IOS) showing that CSD is generated only in the neocortex in a representative coronal slice including the neocortex, the hippocampus, the dorsal striatum, the globus pallidus and the thalamus (Bregma 1.46, Interaural 2.34mm); left, raw image; right, image processing to highlight the CSD wave. The pipette is the LFP extracellular recording electrode. Lower panels: Intrinsic Optical Signal (IOS) showing that CSD is generated only in the neocortex in another representative coronal slice including the neocortex, the dorsal striatum, the globus pallidus and the thalamus (Bregma -0.82, Interaural 2.98mm); left, raw image; right, image processing to highlight the CSD wave. CSD does not propagate to other areas because connections have been severed by slice cutting. Time is indicated as min:sec. 5 images/s. See Figure 1 for details.

Video 2. Classical method of CSD induction in slices: representative CSD triggered in the neocortex by a focal puff of 130mM KCl delivered with a picopump (500ms, 10PSI) visualized by intrinsic optical imaging in raw images (left panel) and after image processing to highlight the wave (right panel). Time is indicated as min:sec. 5 images/s. See Supplementary Figure S3B for details.

Video 3. CSD was readily triggered in the dorsal striatum by a focal puff of 130mM KCl delivered with a picopump (500ms, 10PSI; upper pipette), as shown by a representative CSD visualized by intrinsic optical imaging in raw images (left panel) and after image processing to highlight the CSD wave (right panel). The lower pipette is the LFP extracellular recording electrode. Time is indicated as min:sec. 5 images/s. See Supplementary Figure S3D1 for details.

Video 4. CSD was readily triggered in the hippocampus by a focal puff of 130mM KCl delivered with a picopump (500ms, 10PSI; left pipette), as shown by a representative CSD visualized by intrinsic optical imaging in raw images (left panel) and after image processing to highlight the CSD wave (right panel). The right pipette is the LFP extracellular recording electrode. Time is indicated as min:sec. 5 images/s. See Supplementary Figure S3D2 for details.

Video 5. Optogenetic activation of GABAergic neurons specifically induce CSD in the neocortex. Representative slice from VGAT-ChR2 mice (which express ChR2 selectively in GABAergic neurons)

containing neocortex, hippocampus, dorsal striatum and thalamus, in which CSD was induced only in the neocortex by the illumination with blue light of a complete hemisphere. The optogenetic illumination has been filtered out for clarity and was larger than the imaged area; illumination was stopped at CSD initiation. The pipette is the LFP extracellular recording electrode. Time is indicated as min:sec. 5 images/s. See Figure 3 for details.

Video 6. Optogenetic CSD induction using continuous spatial illumination (illumination area: 1.1mm^2), visualized by intrinsic optical imaging in raw images (left panel) and after image processing (right panel). For better illustrating the experiment, in these images the optogenetic illumination has not been filtered out and was stopped at CSD initiation. Time is indicated as min:sec. 5 images/s.

Video 7. Optogenetic CSD induction using 100ms-5Hz trains of spatial illumination (illumination area: 1.1mm^2), visualized by intrinsic optical imaging in raw images; the optogenetic illumination has not been filtered out for better illustrating the experiment, and was stopped at CSD initiation. CSD has been induced with discontinued illumination in 62.5% of the slices (n = 8 slices in total). Time is indicated as min:sec. The video has been recorded at 5 images/s and is shown at 50 images/s.

Video 8. CSD induction triggered by a focal application of 12mM KCl delivered with a picopump (3PSI, until CSD induction) visualized by intrinsic optical imaging in raw images. Application of KCl is indicated in the video by the “Perfusion” label. Time is indicated as min:sec; 5 images/s.

Video 9. Representative video of double juxtacellular loose patch recordings from a GABAergic neuron and a pyramidal neuron at the site of initiation of CSD, triggered with spatial optogenetic stimulation (which has been filtered out). 5 images/s.

Supplementary references

1. Zerimech S, Chever O, Scalmani P, Pizzamiglio L, Duprat F, and Mantegazza M. Cholinergic modulation inhibits cortical spreading depression in mouse neocortex through activation of muscarinic receptors and decreased excitatory/inhibitory drive. *Neuropharmacology.* 2020;166(107951.
2. Hedrich UB, Liautard C, Kirschenbaum D, Pofahl M, Lavigne J, Liu Y, Theiss S, Slotta J, Escayg A, Dihne M, et al. Impaired action potential initiation in GABAergic interneurons causes hyperexcitable networks in an epileptic mouse model carrying a human Na(V)1.1 mutation. *J Neurosci.* 2014;34(45):14874-89.
3. Tottene A, Conti R, Fabbro A, Vecchia D, Shapovalova M, Santello M, van den Maagdenberg AM, Ferrari MD, and Pietrobon D. Enhanced excitatory transmission at cortical synapses as the basis for facilitated spreading depression in Ca(v)2.1 knockin migraine mice. *Neuron.* 2009;61(5):762-73.
4. Ogiwara I, Iwasato T, Miyamoto H, Iwata R, Yamagata T, Mazaki E, Yanagawa Y, Tamamaki N, Hensch TK, Itohara S, et al. Nav1.1 haploinsufficiency in excitatory neurons ameliorates seizure-associated sudden death in a mouse model of Dravet syndrome. *Hum Mol Genet.* 2013;22(23):4784-804.
5. Yamagata T, Ogiwara I, Tatsukawa T, Otsuka Y, Mazaki E, Inoue I, Tokonami N, Hibi Y, Itohara S, and Yamakawa K. Scn1a-GFP transgenic mouse revealed Nav1.1 expression in neocortical pyramidal tract projection neurons. *bioRxiv.* 2021:2021.03.31.437794.
6. Yu FH, Mantegazza M, Westenbroek RE, Robbins CA, Kalume F, Burton KA, Spain WJ, McKnight GS, Scheuer T, and Catterall WA. Reduced sodium current in GABAergic interneurons in a mouse model of severe myoclonic epilepsy in infancy. *NatNeurosci.* 2006;9(9):1142-9.
7. Dutton SB, Makinson CD, Papale LA, Shankar A, Balakrishnan B, Nakazawa K, and Escayg A. Preferential inactivation of Scn1a in parvalbumin interneurons increases seizure susceptibility. *Neurobiol Dis.* 2013;49(211-20.
8. Westenbroek RE, Merrick DK, and Catterall WA. Differential subcellular localization of the RI and RII Na+ channel subtypes in central neurons. *Neuron.* 1989;3(6):695-704.
9. Gong B, Rhodes KJ, Bekele-Arcuri Z, and Trimmer JS. Type I and type II Na(+) channel alpha-subunit polypeptides exhibit distinct spatial and temporal patterning, and association with auxiliary subunits in rat brain. *JComp Neurol.* 1999;412(2):342-52.
10. Mantegazza M, Cestele S, and Catterall W. Sodium Channelopathies of Skeletal Muscle and Brain. *Physiol Rev.* 2021.
11. Mantegazza M, and Broccoli V. SCN1A/NaV 1.1 channelopathies: Mechanisms in expression systems, animal models, and human iPSC models. *Epilepsia.* 2019;60 Suppl 3(S25-S38.
12. Tang B, Dutt K, Papale L, Rusconi R, Shankar A, Hunter J, Tufik S, Yu FH, Catterall WA, Mantegazza M, et al. A BAC transgenic mouse model reveals neuron subtype-specific effects of a Generalized Epilepsy with Febrile Seizures Plus (GEFS+) mutation. *NeurobiolDis.* 2009;35(1):91-102.
13. Chever O, Djukic B, McCarthy KD, and Amzica F. Implication of Kir4.1 channel in excess potassium clearance: an in vivo study on anesthetized glial-conditional Kir4.1 knock-out mice. *J Neurosci.* 2010;30(47):15769-77.
14. Holthoff K, and Witte OW. Intrinsic optical signals in rat neocortical slices measured with near-infrared dark-field microscopy reveal changes in extracellular space. *J Neurosci.* 1996;16(8):2740-9.
15. Edelstein AD, Tsuchida MA, Amodaj N, Pinkard H, Vale RD, and Stuurman N. Advanced methods of microscope control using muManager software. *J Biol Methods.* 2014;1(2).
16. Schindelin J, Rueden CT, Hiner MC, and Eliceiri KW. The ImageJ ecosystem: An open platform for biomedical image analysis. *Mol Reprod Dev.* 2015;82(7-8):518-29.

17. Bechi G, Scalmani P, Schiavon E, Rusconi R, Franceschetti S, and Mantegazza M. Pure haploinsufficiency for Dravet syndrome Na(V)1.1 (SCN1A) sodium channel truncating mutations. *Epilepsia.* 2012;53(1):87-100.
18. Cestele S, Schiavon E, Rusconi R, Franceschetti S, and Mantegazza M. Nonfunctional NaV1.1 familial hemiplegic migraine mutant transformed into gain of function by partial rescue of folding defects. *Proc Natl Acad Sci U S A.* 2013;110(43):17546-51.
19. Paxinos G, and Watson C. *The Rat Brain in Stereotaxic Coordinates* Massachussets: Elsevier; 2007.
20. Desroches M, Faugeras O, Krupa M, and Mantegazza M. Modeling cortical spreading depression induced by the hyperactivity of interneurons. *J Comput Neurosci.* 2019;47(2-3):125-40.
21. Golomb D, Donner K, Shacham L, Shlosberg D, Amitai Y, and Hansel D. Mechanisms of firing patterns in fast-spiking cortical interneurons. *PLoS Comput Biol.* 2007;3(8):e156.
22. Kager H, Wadman WJ, and Somjen GG. Simulated seizures and spreading depression in a neuron model incorporating interstitial space and ion concentrations. *J Neurophysiol.* 2000;84(1):495-512.
23. Bouret Y, Argentina M, and Counillon L. Capturing intracellular pH dynamics by coupling its molecular mechanisms within a fully tractable mathematical model. *PLoS One.* 2014;9(1):e85449.
24. Huguet G, Joglekar A, Messi LM, Buckalew R, Wong S, and Terman D. Neuroprotective Role of Gap Junctions in a Neuron Astrocyte Network Model. *Biophys J.* 2016;111(2):452-62.
25. Magistretti J, and Alonso A. Biophysical properties and slow voltage-dependent inactivation of a sustained sodium current in entorhinal cortex layer-II principal neurons: a whole-cell and single-channel study. *J Gen Physiol.* 1999;114(4):491-509.
26. Bean BP. The action potential in mammalian central neurons. *Nat Rev Neurosci.* 2007;8(6):451-65.
